# What the Wild Things Do: Mechanisms of Plant Host Manipulation by Bacterial Type III-Secreted Effector Proteins

**DOI:** 10.3390/microorganisms9051029

**Published:** 2021-05-11

**Authors:** Karl J. Schreiber, Ilea J. Chau-Ly, Jennifer D. Lewis

**Affiliations:** 1 Department of Plant and Microbial Biology, University of California, Berkeley, CA 94710, USA; karl.j.schreiber@gmail.com (K.J.S.); ilea_chau@berkeley.edu (I.J.C.-L.); 2 Plant Gene Expression Center, United States Department of Agriculture, University of California, Berkeley, CA 94710, USA

**Keywords:** type III secreted effector, biochemical activity, *Pseudomonas syringae*, *Ralstonia*, *Xanthomonas*, virulence promotion, effector-triggered immunity, host activation, host targets

## Abstract

Phytopathogenic bacteria possess an arsenal of effector proteins that enable them to subvert host recognition and manipulate the host to promote pathogen fitness. The type III secretion system (T3SS) delivers type III-secreted effector proteins (T3SEs) from bacterial pathogens such as *Pseudomonas syringae*, *Ralstonia solanacearum*, and various *Xanthomonas* species. These T3SEs interact with and modify a range of intracellular host targets to alter their activity and thereby attenuate host immune signaling. Pathogens have evolved T3SEs with diverse biochemical activities, which can be difficult to predict in the absence of structural data. Interestingly, several T3SEs are activated following injection into the host cell. Here, we review T3SEs with documented enzymatic activities, as well as T3SEs that facilitate virulence-promoting processes either indirectly or through non-enzymatic mechanisms. We discuss the mechanisms by which T3SEs are activated in the cell, as well as how T3SEs modify host targets to promote virulence or trigger immunity. These mechanisms may suggest common enzymatic activities and convergent targets that could be manipulated to protect crop plants from infection.

## 1. Introduction

The outcome of plant–pathogen interactions is determined by a complex network of molecular events that involve proteins and other macromolecules from both the host and pathogen. The presence of a potential pathogen in host tissues is first detected in the extracellular space by plant pattern recognition receptors (PRRs) which recognize conserved molecular signatures known as pathogen-associated molecular patterns (PAMPs). Two of the most well-studied PAMPs are bacterial flagellin and translational elongation factor Tu, which are recognized by the receptor-like kinase (RLK) PRRs FLAGELLIN-SENSING 2 (FLS2) and ELONGATION FACTOR RECEPTOR (EFR), respectively [1,2,3]. Following PAMP recognition, PRRs associate with additional transmembrane RLKs such as BRI1-ASSOCIATED RECEPTOR KINASE1 (BAK1) as well as receptor-like cytoplasmic kinases (RLCKs) to transduce an intracellular signal via mitogen-activated protein (MAP) kinase cascades for the activation of PAMP-triggered immunity (PTI) [4,5,6]. This immune response involves transcriptional reprogramming, callose deposition in the cell wall, production of reactive oxygen species (ROS), and secretion of antimicrobial compounds, all of which serve to prevent an infection from becoming established. Faced with this defensive barrier, many phytopathogenic bacteria have evolved virulence effector proteins that are introduced into host cells via a needle-like structure known as a type III secretion system [7]. These type III-secreted effectors (T3SEs) are targeted to multiple locations in the host intracellular space where they act to suppress host immune responses and restore bacterial virulence [8,9,10]. Depending on the genetic background of the host, the advantage conferred by T3SEs can become a liability, as plants have evolved intracellular nucleotide-binding leucine rich repeat (NLR) proteins to recognize T3SEs, either by direct interaction or by detecting the enzymatic activity of the T3SE [11,12]. Indirect mechanisms of T3SE recognition may involve an NLR “guarding” a virulence target and monitoring for T3SE-mediated modifications to its “guardee”, or the NLR may interact with a “decoy” protein that mimics a true virulence target in order to detect T3SE activity [13]. Some NLRs have evolved as translational fusions with decoy proteins, referred to as “integrated domains”. Recognition of a T3SE elicits a more intense immune response (effector-triggered immunity, or ETI) that may culminate in a localized programmed cell death response known as a hypersensitive response (HR), ultimately restricting the spread of an infection in host tissues. The evolution of additional T3SEs that interfere with ETI in turn drives further NLR evolution, resulting in a dynamic molecular interface between plants and prospective pathogens. Beyond the PRR and NLR surveillance systems, plants have also evolved a complex signaling network to coordinate appropriate immune responses, mediated by phytohormones such as salicylic acid (SA), jasmonic acid (JA), ethylene, abscisic acid, auxins, cytokinins, gibberellins, and brassinosteroids [14]. Furthermore, elicitation of defense responses locally at the site of infection may stimulate the release of additional mobile signaling molecules that reduce the susceptibility of distal tissues to infection, in a phenomenon known as systemic acquired resistance (SAR) [15,16]. Altogether, the signaling infrastructure of plant immunity provides numerous targets for potential virulence-promoting T3SEs. 

**Figure 1:**
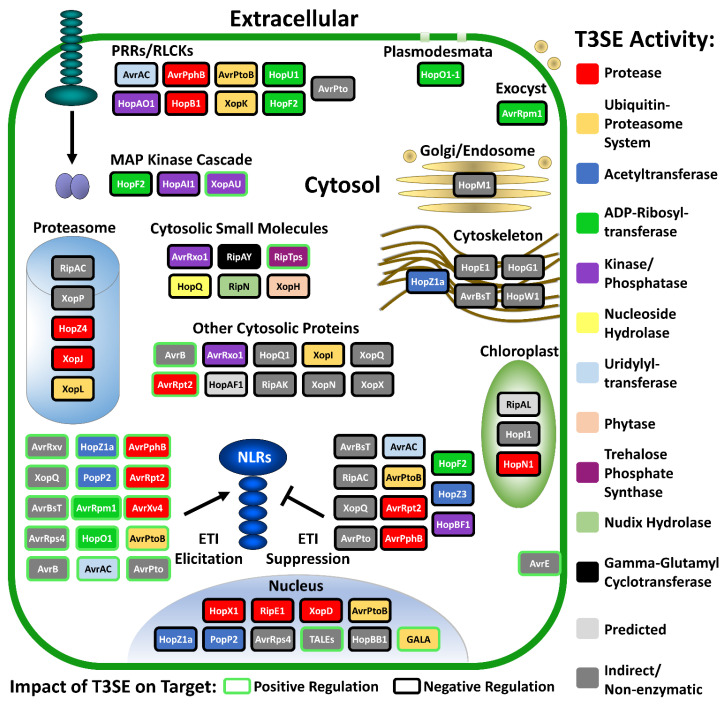
Schematic overview of bacterial type III-secreted effector (T3SE) activities in the plant intracellular space. The T3SEs discussed in this review are arranged in functional categories based on their characterized target(s) in terms of biological process or subcellular location. For each T3SE, a black border indicates that the T3SE negatively regulates its target in some manner, while T3SEs with a green border function as positive regulators. Note that the GALA family of T3SEs interacts with several target proteins that are primarily nuclear-localized but may be found in other subcellular locations as well. Limited data are available for the localization of AvrE-type T3SEs, but AvrE1 is known to associate with the plasma membrane.

One of the most remarkable aspects of bacterial T3SEs is that they have evolved in prokaryotic cells as tools that must be functional in eukaryotic cells. This requires the T3SE to be correctly localized within the host cell, to interact with substrates that may be different from any endogenous bacterial proteins, and to provide adequate enzymatic activity in the eukaryotic intracellular milieu. In this review, we provide a survey of T3SEs from phytopathogenic bacteria with a particular emphasis on the enzymatic activities utilized by these proteins to promote pathogen virulence. We also note instances where this activity is known to stimulate NLR-mediated ETI. Our focus is largely on T3SEs with documented enzymatic activities, although we also discuss T3SEs that facilitate virulence-promoting processes either indirectly or through non-enzymatic mechanisms. Where appropriate, we consider the evolutionary origins of different T3SE activities and enumerate the host-mediated modifications of certain T3SEs that are required for their function in host cells. Cumulatively, these details reveal a striking convergence in both the enzymatic activities and host targets of T3SEs in plant cells (summarized in Figure 1 and Table 1).

## 2. T3SEs with Protease Activity

One effective and widely adopted pathogen virulence strategy involves the elimination of key plant immune regulators by proteolytic cleavage. Protease enzymes cleave peptide bonds within proteins and are classified into seven broad groups based on their catalytic residues as aspartic, cysteine, glutamic, serine, threonine, or metalloproteases, in addition to asparagine peptide lyases [122,123]. These enzymes are distributed throughout all kingdoms of life, which provides a feedstock for T3SE evolution from endogenous bacterial proteases, interbacterial horizontal gene transfer, and gene acquisition from non-bacterial sources. The wide diversity of known protease substrates also implies that proteolytic T3SEs can be highly versatile tools with which pathogens can influence host immunity.

### 2.1. T3SEs with Cysteine Protease Activity—AvrRpt2, AvrPphB, HopN1, HopX1, RipE1, XopJ, HopZ4, XopD, and AvrXv4

One of the most well-characterized proteolytic T3SEs is AvrRpt2, which was initially identified in *P. syringae* pv. *tomato* JL 1065 as an elicitor of the HR on certain Arabidopsis ecotypes and soybean (*Glycine max*) cultivars [124]. Subsequent genetic analysis pinpointed RESISTANCE TO PSEUDOMONAS SYRINGAE 2 (RPS2) as the NLR responsible for AvrRpt2 recognition [125]. This recognition is not direct, however, but involves a complex of RPS2 with the immune regulator RPM1-INTERACTING PROTEIN 4 (RIN4), and RPS2 is activated by the AvrRpt2-mediated elimination of RIN4 [25,126,127]. For functional characterization, the predicted secondary structure of AvrRpt2 was found to be similar to the solved crystal structures of some cysteine protease enzymes, including the presence of a putative catalytic triad (cysteine, histidine, aspartic acid) in AvrRpt2 [126]. The catalytic triad is required both for the elimination of RIN4 by AvrRpt2 and for recognition of this T3SE by RPS2. Interestingly, RIN4 is also used by the NLR RESISTANCE TO PSEUDOMONAS SYRINGAE PV. MACULICOLA 1 (RPM1) to recognize the T3SEs AvrB and AvrRpm1, so the presence of AvrRpt2 can interfere with RPM1-mediated ETI [41,42,43,128]. In the absence of RPS2, cleavage of RIN4 by AvrRpt2 facilitates suppression of PTI through a yet-undetermined mechanism, albeit one which is dependent on the catalytic triad of AvrRpt2 [89,129]. Some degree of virulence-promoting activity for AvrRpt2 is still detectable in a *rin4* background, suggesting that there are additional AvrRpt2 virulence targets [130,131]. Indeed, RIN4 belongs to a diverse family of nitrate-induced (NOI) domain-containing proteins, and several other members of this family can be cleaved by AvrRpt2, with a consensus cleavage site of [LVI] PxFGxW [132,133]. One notable RIN4-independent AvrRpt2 activity is the suppression of flg22-induced activation of the MAPKs MPK4 and MPK11 [133]. The role of NOI family proteins in this process is unclear, however, as individual knockouts of NOI protein genes did not alter the MPK suppression phenotype, although not all NOI protein genes were tested and functional redundancy could be a complicating issue. More generally, the NOI protein family is largely uncharacterized aside from the presence of conserved PxFGxW and Y/FTxxF motifs, and despite their name, NOI domain-containing proteins are not involved in nitrogen metabolism [134]. Nonetheless, the discovery of AvrRpt2 orthologs in diverse bacterial species, capable of cleaving RIN4 and other NOI proteins, suggests that AvrRpt2 is an important contributor to bacterial virulence. 

The T3SE AvrPphB (or hopAR1) was also first described as an avirulence determinant, based on the ability of a protein from *P. syringae* pv. *phaseolicola* 1301A to elicit an HR on specific cultivars of bean (*Phaseolus vulgaris*) [135]. Sequence analyses revealed that AvrPphB was similar to the YopT T3SE family from various *Yersinia* species and highlighted a conserved cysteine/histidine/aspartic acid triad typically found in cysteine proteases [136]. Indeed, AvrPphB is proteolytically processed in *Pseudomonas* prior to secretion [137], and this cleavage is dependent on the cysteine/histidine/aspartic acid triad, indicative of an autocatalytic mechanism [136]. The avirulence function of AvrPphB also requires the catalytic triad. The recognition of AvrPphB involves the NLR RESISTANCE TO PSEUDOMONAS SYRINGAE 5 (RPS5) [138] in complex with the RLCK AVRPPHB SUSCEPTIBLE 1 (PBS1) [39,139,140]. Cleavage of PBS1 by AvrPphB elicits RPS5-mediated ETI, although the activation of RPS5 likely involves sensing a general conformational change in PBS1 rather than the generation of free protein ends resulting from cleavage [141]. The activity of AvrPphB as a protease is further evidenced by observations that PBS1 cleavage requires an intact catalytic triad in AvrPphB [40] and that this cleavage is largely blocked by incubation with the protease inhibitor E-64 [142]. Additionally, the AvrPphB crystal structure closely resembles structures of other cysteine proteases [142]. In terms of virulence function, AvrPphB suppresses PTI-associated gene expression and callose deposition in a mostly PBS1-independent manner [37]. A survey of PBS1-like (PBL) proteins revealed that AvrPphB cleaves additional RLCKs including BOTRYTIS-INDUCED KINASE 1 (BIK1) and two PBLs designated PBL1 and PBL2. These RLCKs interact with and act directly downstream of PRRs such as FLS2 and EFR, so their cleavage by AvrPphB compromises PTI and promotes pathogen proliferation. Likewise, signaling downstream of the chitin receptor CERK1 is predicted to be disrupted by AvrPphB-mediated cleavage of PBL27 [143,144]. Effector-triggered immunity is also impacted by AvrPphB, specifically the recognition of the T3SE AvrB by the NLR RPM1. This recognition occurs when AvrB associates with RPM1-INTERACTING PROTEIN KINASE (RIPK), which phosphorylates the immune regulator RIN4 and thus activates RPM1 [89,90]. The cleavage of RIPK by AvrPphB blocks the recognition of AvrB to restore the virulence of strains carrying this T3SE [38]. Overall, the sequences of known AvrPphB substrates reveal that cleavage occurs C-terminal to a moderately conserved GDK motif, although additional residues are involved in substrate engagement, as the GDK sequence is necessary but not sufficient for cleavage [37,40,142].

In contrast to the broad range of putative substrates for AvrRpt2 and AvrPphB, the T3SE HopN1 appears to act on one specific target. Initial functional characterization of HopN1 determined that this T3SE was capable of suppressing necrotic disease symptoms on susceptible hosts and inhibiting nonhost HRs [145]. A predicted cysteine protease catalytic triad was verified as being essential for these suppressive phenotypes, and cysteine protease activity was demonstrated in vitro with purified recombinant HopN1 and resorufin-labeled casein as a generic protease substrate [145]. Purified HopN1 was also used for an in vitro pull-down from tomato leaf extract, which identified PsbQ, a member of the oxygen-evolving complex of photosystem II, as a HopN1 interactor [44]. The interaction was validated in vitro, and interestingly, a catalytic mutant of HopN1 did not bind PsbQ. Notably, HopN1 localizes to chloroplasts and reduces the electron transport activity of photosystem II [44]. Proteolysis of PsbQ was observed when thylakoid samples were incubated with partially purified HopN1, again dependent on the catalytic triad [44]. PsbQ-silenced tobacco plants exhibit reduced ROS production following exposure to a virulent pathogen, as well as diminished nonhost HRs [44]. Although a direct effect of HopN1 on bacterial growth could not be detected in planta, it is possible that HopN1 reduces ROS accumulation during infection and could suppress cell death responses elicited by other T3SEs. 

Proteolytic T3SEs may also target a specific family of proteins, as illustrated by HopX1 from *P. syringae* pv. *tabaci* 11528 and RipE1 from *R. solanacearum*. For HopX1, it was known that *P. syringae* pv. *tabaci* 11528 lacks coronatine but can still overcome PTI-associated stomatal closure, suggesting that this strain might possess another virulence factor that mimics coronatine as a strategy for modulating stomatal aperture [45,146]. Coronatine facilitates the interaction of CORONATINE-INSENSITIVE 1 (COI1) and JASMONATE-ZIM DOMAIN (JAZ) proteins, which results in JAZ degradation and de-repression of jasmonate signaling [147,148]. With this in mind, Gimenez-Ibanez et al. [45] conducted a screen to identify T3SEs from *P. syringae* pv. *tabaci* 11528 capable of destabilizing JAZ proteins. They noted that HopX1 possessed such activity, and that the amino acid sequence of this T3SE included a cysteine protease-like cysteine/histidine/aspartic acid catalytic triad. Proteolytic activity was confirmed in vitro using JAZ5 as a substrate, and HopX1 activity was shown to be sensitive to protease inhibitors [45]. In planta, JAZ1, JAZ2, JAZ5, JAZ9, and JAZ10 all interact with and are degraded by HopX1, and degradation requires the HopX1 catalytic triad [45]. Ultimately, JAZ protein degradation induces jasmonate-responsive gene expression, which suppresses SA-dependent gene expression and enhances the susceptibility of Arabidopsis plants to infection by *P. syringae*. Similarly, RipE1 was identified in a screen for *R. solanacearum* T3SEs capable of suppressing a flg22-induced ROS burst [47]. Subsequent characterization in yeast two-hybrid experiments indicated that RipE1 interacts with JAZ4, JAZ9, and JAZ10. These three proteins are degraded by RipE1 in planta, and a cysteine protease-like catalytic triad within RipE1 is required for this degradation [47]. As with HopX1, JAZ degradation by RipE1 increases host susceptibility to infection by suppressing SA-mediated gene expression. Despite the similar enzymatic activities of HopX1 and RipE1, only RipE1 can suppress flg22-induced ROS accumulation and PTI-related gene expression [47], indicating some mechanistic differences that remain to be explored.

Another avenue for the manipulation of phytohormone signaling involves proteolysis of components of the proteasome, which is a key mediator of protein turnover. The *Xanthomonas euvesicatoria* T3SE XopJ and *P. syringae* T3SE HopZ4 are both members of the widely distributed and highly diverse YopJ superfamily [149]. Although several members of this family exhibit acetyltransferase activity (see Section 5), the conserved catalytic site of these enzymes (histidine, aspartic acid/glutamic acid, cysteine) resembles that of cysteine proteases, and weak protease activity can be detected for some acetyltransferase T3SEs [18,150]. Importantly, the catalytic triad is conserved in both XopJ and HopZ4 [46,48,151]. Functionally, XopJ directly interacts with and degrades a subunit of the host proteasome, REGULATORY PARTICLE TRIPLE-A ATPASE 6 (RPT6) [48,151]. This degradation is impaired by mutation of the putative catalytic cysteine and by treatment with the cysteine protease inhibitor E-64, suggesting that the disappearance of RPT6 is due to XopJ-mediated proteolysis. RPT6 degradation inhibits the proteasome and impairs the turnover of NONEXPRESSOR OF PATHOGENESIS-RELATED GENES 1 (NPR1), a major regulator of SA-dependent defense responses [48,151]. Accordingly, *X. euvesicatoria* Δ*xopJ* mutants trigger early necrosis in susceptible pepper plants, but not in pepper plants with reduced expression of NPR1, indicating that XopJ interferes with SA responses in order to delay the onset of necrosis [151]. This likely has many additional downstream effects, as supported by the suppression of callose deposition and other defense responses in transgenic Arabidopsis plants expressing XopJ [49]. Interference with SA signaling also plays a role in XopJ avirulence activity, as XopJ triggers an HR only upon application of SA in *N. benthamiana* and this HR is abolished when *NPR1* is silenced [152]. Similar to XopJ, HopZ4 was also shown to interact with RPT6 in yeast and in planta [46]. Moreover, HopZ4 was shown to suppress proteasome activity in a manner dependent on an intact catalytic triad, suggesting it also acts as a protease to degrade RPT6 and could similarly affect SA signaling [46].

Cysteine protease T3SEs can also target specific post-translational modifications in host proteins, as illustrated by the *X. euvesicatoria* T3SE XopD [153]. An examination of the XopD amino acid sequence indicated that the C-terminus of this T3SE is similar to the C48 family of cysteine proteases, which contains enzymes that deconjugate small ubiquitin-like modifier (SUMO) moieties from protein substrates [51]. Notably, XopD was found to be homologous to the ubiquitin-like, de-SUMOylating protease Ulp1, including the conservation of catalytic residues (histidine, aspartic acid, cysteine) and residues from the Ulp1 crystal structure that are important for substrate recognition. Determination of the XopD crystal structure later confirmed the homology with Ulp1 [154]. In vitro assays with purified XopD and a ^35^S-tomato SUMO-HA substrate demonstrated that XopD can proteolytically cleave SUMO precursor proteins [51]. Similar to other cysteine proteases from the C48 family, XopD SUMO protease activity was blocked by the protease inhibitors iodoacetamide and N-ethylmaleimide, as well as by mutation of the catalytic residues [51]. Similarly, XopD exhibits isopeptidase activity to deSUMOylate proteins both in vitro and in planta, which is also dependent on the protease catalytic residues. Functionally, XopD inhibits the expression of genes associated with ethylene biosynthesis in tomato, resulting in enhanced susceptibility to *X. euvesicatoria*, yet reduced development of chlorotic disease symptoms [52,155]. This activity is facilitated by the association of XopD with the transcription factor *SOLANUM LYCOPERSICON* ETHYLENE RESPONSE FACTOR 4 (SlERF4) [52]. This transcription factor is mono-SUMOylated in planta, and the removal of this modification by XopD destabilizes SlERF4 and impairs the expression of SlERF4-activated genes [52]. These deSUMOylation events are dependent on XopD protease activity but are also mitigated by the proteasome inhibitor MG132 [52]. In addition, XopD contains an ERF-associated amphiphilic repression (EAR) motif that contributes significantly to SlERF4 deSUMOylation, independent of any role as a simple binding interface. Interestingly, XopD also activates the expression of a putative basic helix–loop–helix transcription factor (bHLH132) in tomato through a yet-unknown mechanism, although this induction is also dependent on XopD SUMO protease activity [156]. In this case, however, bHLH132 expression is associated with host defense activation, indicating a potential liability to the virulence function of XopD arising from “off-target” T3SE activity. *X. euvesicatoria* possesses another T3SE, AvrXv4, whose sequence includes a conserved SUMO protease-like catalytic triad [50]. Enzymatic activity could not be detected in vitro, but transient expression of AvrXv4 in planta reduced the accumulation of co-expressed SUMO substrates in a catalytic site-dependent manner, suggesting that a plant co-factor is required [50]. Functionally, AvrXv4 elicits an HR in *N. benthamiana* via an unidentified NLR, and this phenotype is dependent on the catalytic residues of AvrXv4 [50]. *X. euvesicatoria* Δ*avrXv4* deletion mutants exhibit slightly reduced growth in tomato leaves, indicating that AvrXv4 does promote pathogen virulence in the absence of ETI, although the host targets of this T3SE are currently unknown [50].

### 2.2. T3SEs with Threonine Protease Activity—HopB1

While the majority of documented T3SE proteases are members of the cysteine protease family, the *P. syringae* T3SE HopB1 belongs to a relatively small family of threonine proteases. This T3SE was initially examined due to its ability to suppress flg22-induced expression of the PTI marker gene *FRK1* [53]. In vitro pull-down assays identified FLS2 as a direct interactor with HopB1, and further in planta assays indicated that this interaction occurs regardless of the presence or absence of flg22 [53]. While HopB1 does not affect the abundance of FLS2, BAK1 disappears rapidly in flg22-treated protoplasts when HopB1 is present. The phosphorylation of BAK1 is essential for this event, indicating that HopB1 only targets activated BAK1. The disappearance of BAK1 results from the cleavage of this protein within the kinase P-loop region, between amino acids arginine-297 and glycine-298 [53]. Three other BAK1-related SOMATIC-EMBRYOGENESIS RECEPTOR-LIKE KINASEs (SERKs) are also cleaved by HopB1 between the same residues [53]. Given that neither the sequence nor predicted structure of HopB1 resembles any other known proteases, the sensitivity of HopB1 to class-specific protease inhibitors was assessed. Only serine protease inhibitors (PMSF and AEBSF) significantly reduced HopB1 protease activity [53]. Subsequent systematic mutagenesis of serine and threonine residues in HopB1 identified threonine-370 as a critical catalytic residue, and additional mutagenesis suggested a putative catalytic quartet (threonine, histidine, aspartic acid, aspartic acid) that is important for PTI suppression. Based on the composition of these catalytic residues, HopB1 is now classified as a threonine protease [123]. Overall, the model of HopB1 function posits that flg22 stimulates recruitment of BAK1 to FLS2, whereupon BAK1 is phosphorylated and subsequently cleaved by HopB1 to block downstream PTI responses [53].

## 3. T3SE Manipulation of the Ubiquitin–Proteasome System

The ubiquitin–proteasome system (UPS) in plants plays a critical role in mediating biotic stress responses not only through the removal of misfolded and defective proteins but also through the regulation of protein stability [157,158]. Proteins destined for degradation by the UPS are ubiquitinated by a cascade of three enzymatic reactions: (1) ubiquitin activation by the E1 enzyme; (2) transfer to the ubiquitin-conjugating enzyme, E2, and (3) attachment to the substrate by the E3 ligase [159]. E3 ligases confer specificity to the UPS through target protein selection [160]. As a result, plants encode a large number of E3 ligases, many of which have been shown to function as regulators of plant immunity and hormone signaling [161,162]. Given the importance of the UPS in regulating immune responses, it follows that pathogens have evolved T3SEs to manipulate the UPS machinery in order to promote virulence during plant–pathogen interactions.

### 3.1. T3SEs That Mimic Eukaryotic E3 Ligases—AvrPtoB, GALAs, and XopI

Several T3SEs have evolved to exploit the host UPS for the targeted degradation of host proteins by exhibiting E3 ubiquitin ligase activity. Perhaps the most well-characterized example of this is the T3SE AvrPtoB (also known as HopAB2) from *P. syringae* pv. *tomato* DC3000. AvrPtoB is a modular protein with two distinct subdomains [163]. The N-terminal region acts to suppress basal immunity and plays a key role in interactions with host proteins [63,64,164]. Meanwhile, the C-terminal domain displays structural homology to eukaryotic U-box/RING E3 ligases and has demonstrated ubiquitin ligase activity, initially confirmed by immunodetection of in vitro autoubiquitinated AvrPtoB [165]. Ubiquitination of host proteins by AvrPtoB has been shown to facilitate the subversion of multiple layers of plant immunity. In Arabidopsis, AvrPtoB catalyzes the ubiquitination and programmed degradation of several components of PTI, including the PRRs EFR, FLS2, BAK1, and CERK1 [63,64,65]. This degradation allows *P. syringae* to overcome PRR-mediated immunity, as evidenced by the reduced growth of *P. syringae* Δ*avrPtoB* knockouts in Arabidopsis, which is not observed in *cerk1* mutants [64]. In addition to suppressing PTI, AvrPtoB E3 ligase activity also interferes with SAR via the degradation of NPR1, a master regulator of SA signaling [66]. In tomato, AvrPtoB can be recognized by the NLR Prf, which interacts with the kinase decoys Fen or Pto [67]. For a Prf–Fen complex, the C-terminal E3 ligase activity of AvrPtoB serves to suppress ETI. This is mediated by an interaction between the kinase Fen and an N-terminal region of AvrPtoB, leading to Fen ubiquitination and proteasome-mediated degradation [67]. Importantly, AvrPtoB was initially discovered as an interactor with the tomato cytosolic kinase Pto [97]. In contrast to Fen, which only interacts with one domain of AvrPtoB, Pto interacts with a second AvrPtoB site distal to the E3 ligase domain [68]. The interaction of a Prf/Pto complex with this distal site in AvrPtoB results in ETI [69]. This demonstrates the role of AvrPtoB not only in promoting virulence but also in triggering immunity in the presence of a cognate NLR.

Similar to AvrPtoB, the highly conserved GALA family of T3SEs in *R. solanacearum* manipulates the host UPS machinery by mimicking eukaryotic E3 ubiquitin ligases, specifically components of SCF-type E3 ligases. SCF-type E3 ligases are multi-unit complexes consisting of SKP1, Cullin1, and F-box protein subunits. Sequence analysis of GALA proteins revealed that each of these T3SEs possesses a 48aa domain characteristic of eukaryotic F-box proteins [70]. Further, GALA proteins were found to directly interact with Arabidopsis SKP1-like proteins in a yeast two-hybrid screen, suggesting that GALAs can be incorporated into the host SCF complex [70]. Although single GALA knockouts do not have a significant effect on *R. solanacearum* virulence, mutants lacking all seven GALA proteins demonstrate significantly attenuated disease symptoms in Arabidopsis and tomato [70,71]. While specific targets have not yet been identified, this suggests a semi-redundant role for the GALA proteins, likely involving the ubiquitination of host defense-related proteins via SCF-type E3 ligase activity. More recently, the T3SE XopI from *Xanthomonas oryzae* pv. *oryzae* has also been identified as an F-box protein that contributes to bacterial virulence in rice. Yeast two-hybrid screens revealed that XopI interacts with the SKP1-like protein OSK1 through its F-box domain and with the thioredoxin OsTrxh2, a positive regulator of rice immunity, through its C-terminal domain [75]. As a result of these interactions, OsTrxh2 is degraded in a UPS-dependent manner. Degradation of OsTrxh2 further leads to the suppression of OsNPR1-dependent signaling, suggesting a role for XopI in suppressing SAR in rice [75].

### 3.2. T3SEs That Possess Novel E3 Ligase Domains—RipAR, RipAW, RipV2, XopK, XopL, and XopAE

Beyond mimicking eukaryotic E3 ligases, there is mounting evidence that bacteria have also evolved novel E3 ubiquitin ligase domains in order to leverage the host UPS. For example, the *R. solanacearum* T3SEs RipAR, RipAW, and RipV2 do not share homology with any known eukaryotic E3 ubiquitin ligases but instead possess a novel E3 ubiquitin ligase (NEL) domain from the IpaH family of *Salmonella* T3SEs [72,73,166]. Notably, RipAR, RipAW, and RipV2 all demonstrate E3 ubiquitin ligase activity in vitro and can suppress PTI responses in *Nicotiana benthamiana* [72], although the specific targets of these three T3SEs remain to be identified. The T3SE XopK from *X. oryzae* pv. *oryzae* also lacks significant similarity to any known E3 ubiquitin ligase but was observed to mediate the proteasome-dependent degradation of OsSERK2, a key regulator of rice immunity [76]. The E3 ubiquitin ligase activity of XopK was confirmed in vitro and shown to be necessary for full pathogen virulence. 

Similarly, the T3SE XopL from *X. euvesicatoria* possesses E3 ubiquitin ligase activity but shows no structural similarity to known E3 ubiquitin ligases [77]. The crystal structure of XopL revealed an E3 ligase domain with a novel fold (termed the XL-domain), which was not present in any previously characterized E3 ubiquitin ligases. Additionally, XopL interacts with specific plant E2 enzymes, suggesting that this T3SE can utilize the existing host machinery for ubiquitination [77]. Recently, a yeast two-hybrid screen identified the autophagy component SH3P2 as a potential interactor with XopL [78]. Further, in vitro ubiquitination assays demonstrated that XopL can ubiquitinate SH3P2. Transient co-expression of XopL and SH3P2 in *N. benthamiana* reduced the abundance of SH3P2 and suppressed autophagy in planta [78]. This suppression was dependent on the proteasome as well as the E3 ligase domain of XopL, indicating that XopL ubiquitinates SH3P2 to target it for degradation by the UPS [78]. SH3P2 was previously found to associate with proteins that play a role in the later stages of autophagy mediated by NEIGHBOR OF BRCA 1 (NBR1) [167,168]. In mammalian systems, NBR1-mediated autophagy plays a key role in immunity by targeting pathogens to the autophagosome for degradation [169]. Plant homologs of NBR1 also influence immunity, indicating that XopL may promote *X. euvesicatoria* virulence by disrupting NBR1-mediated autophagic degradation. Interestingly, XopL undergoes autoubiquitination in planta, and ubiquitinated XopL is targeted for NBR1-mediated autophagic degradation [78]. Taken together, these data suggest XopL may undergo self-modification in order to access the host autophagy machinery and ultimately suppress host degradation mechanisms by targeting SH3P2. Following the identification of the novel XL-domain in XopL, another XL-domain-possessing T3SE, XopAE, was reported in *X. euvesicatoria* [74]. As seen with XopL, ubiquitination assays indicated that XopAE displays E3 ubiquitin ligase activity in vitro. Furthermore, this ligase activity was required for XopAE inhibition of PTI signaling, suggesting XopAE may target components of PTI for degradation [74].

## 4. ADP-Ribosyltransferase T3SEs

The ADP-ribosyltransferases (ADP-RTs) comprise a structurally diverse family of enzymes that are distributed throughout all kingdoms of life [170,171]. These enzymes catalyze the reversible transfer of ADP-ribose from nicotinamide adenine dinucleotide (NAD^+^) to proteins or other macromolecules [172,173,174]. Within this family, mono(ADP-ribosyl)transferases (mADP-RTs) attach a single ADP-ribose to their substrates, while poly(ADP-ribosyl)transferases (pADP-RTs) transfer multiple ADP-ribose moieties. The first identified mADP-RTs were bacterial in origin and included diphtheria toxin and cholera toxin, which are key mediators of pathogen virulence in mammalian hosts [175,176]. More recently, endogenous pADP-RT activity in Arabidopsis was shown to regulate host gene expression during immune responses and significantly influence susceptibility to pathogen infection [177,178,179]. The manipulation of host protein ADP-ribosylation thus represents a potentially important virulence target for T3SEs.

### T3SEs with mADP-RT Activity—HopF2, HopU1, HopO1-1, and AvrRpm1

The first hint that phytopathogens might use ADP-ribosylation to subvert host immunity was derived from the crystal structure of the T3SE HopF1 (formerly AvrPphF) from *P. syringae* pv. *phaseolicola* 1449B [180]. A subdomain of this protein is structurally similar to the catalytic domain of diphtheria toxin and includes conserved surface-exposed residues (arginine-72, aspartic acid-174) that were shown to contribute to the virulence function of HopF1 in bean plants. Subsequent advances in the functional characterization of this T3SE family have come from HopF2, a related allele from *P. syringae* pv. *tomato* DC3000. In Arabidopsis, HopF2 suppresses numerous PAMP-induced responses including callose deposition, ROS bursts, MAPK activation, and expression of PTI marker genes [29,30]. The effect on marker gene expression was abolished by mutation of the conserved surface-exposed residues arginine-71 and aspartic acid-175. Furthermore, HopF2 interacts with the MAP kinase kinases (MKKs) MKK4 and MKK5 [29]. For MKK5, this prevents the key autophosphorylation event in MKK5 activation, blocking the downstream phosphorylation of MAPK6 and activation of PTI. The ADP-RT activity of HopF2 was demonstrated in vitro using MKK5 as a substrate in combination with biotinylated NAD as an ADP-ribose donor, followed by immunodetection of biotinylated substrates [29]. While the detected ADP-RT activity was arginine-71/aspartic acid-175-dependent, R71A and D175A substitutions eliminated the HopF2–MKK5 interaction, suggesting that these residues may comprise an important interaction interface rather than participate in reaction catalysis [29]. Further upstream in the PTI signaling pathway, HopF2 blocks the phosphorylation of BIK1, PBL1, and PBS1, which are RLCKs that interact with PRRs and transduce signals for immune activation [31]. This blockage is accomplished by the interaction of HopF2 with the transmembrane and kinase domains of the PRR-associated protein BAK1, although direct ADP-ribosylation remains to be demonstrated. It is hypothesized that this modification would interfere with the ability of BAK1 to activate downstream RLCKs and thereby disrupt the signaling cascade from MKK1/MKK2 to MAPK4 to PTI execution.

In addition to PTI, HopF2 also ADP-ribosylates host proteins involved in ETI. Wilton et al. [28] observed that bacterial expression of HopF2 can block the HR elicited in Arabidopsis by the cysteine protease T3SE AvrRpt2. Further investigation revealed that HopF2 interacts with and ADP-ribosylates RIN4 to prevent the AvrRpt2-mediated degradation of RIN4 that would normally elicit ETI [28,29]. These phenotypes were not observed in a HopF2 D175A mutant. Beyond interfering with ETI mediated by other T3SEs, the ADP-RT activity of a HopF2 allele (HopF1r) from *P. syringae* pv. *aceris* M302273PT can be recognized by the NLR HOPZ ACTIVATED RESISTANCE 1 (ZAR1) in combination with the RLCK ZED1-RELATED KINASE 3 (ZRK3) [181]. As HopF2a does not ADP-ribosylate ZRK3, there is likely another protein in the ZAR1–ZRK3 complex that senses this enzymatic activity prior to the activation of ETI.

In contrast to HopF2, HopU1 was first posited as an ADP-RT based on sequence analyses that highlighted conserved catalytic residues (R-ST-E) that are also found in cholera toxin [35]. An in vitro assay using ^32^P-labeled NAD and the generic substrate poly-L-arginine confirmed that HopU1 possesses ADP-RT activity [35]. Proteomic screening identified two glycine-rich RNA-binding proteins, GRP7 and GRP8, as HopU1 substrates that are ADP-ribosylated at two sites in their RNA recognition motifs [35]. Both GRP7 and GRP8 bind to the 3′UTR of mRNAs encoding the PRRs FLS2 and EFR, and ADP-ribosylation by HopU1 reduces the binding affinity of GRP7/8 for these transcripts [36,182]. The resulting impairment of FLS2/EFR translation attenuates PRR accumulation in response to infection and reduces host PTI induction. The crystal structure of HopU1 exhibits little similarity to that of HopF1, and HopU1 more closely resembles cholera toxin versus the diphtheria toxin-like structure of HopF1 [182].

The T3SE HopO1-1 was also identified as a putative ADP-RT based on a search for conserved amino acid sequence motifs, which indicated that HopO1-1 and HopU1 share the same conserved catalytic residues [32,35]. The enzymatic activity of purified recombinant HopO1-1 was verified using poly-L-arginine as an ADP-ribosylation substrate and shown to be dependent on the predicted catalytic residues [32]. Mechanistically, HopO1-1 interacts with the plasmodesmata-localized proteins PDLP5 and PDLP7, causing their proteasome-dependent degradation in vitro [32]. In planta, only PDLP7 was destabilized by HopO1-1 and thus likely represents the biologically relevant target. Although direct ribosylation of PDLP7 could not be demonstrated, mutation of two arginine residues in the HopO1-1-binding C-terminal region of PDLP7 prevented the degradation of this protein, presumably by blocking its ADP-ribosylation by HopO1-1 [32]. Overall, HopO1-1 is thought to destabilize PDLP7 to enhance molecular flux through plasmodesmata and facilitate the spread of bacteria in infected tissues. Interestingly, the NLR ZAR1 in complex with ZRK3 recognizes a HopO1 allele and elicits ETI in a manner dependent on the catalytic sites of the T3SE [33,34]. As with HopF1r, the direct ADP-ribosylation substrate for HopO1 recognition remains to be identified.

The *P. syringae* T3SE AvrRpm1 was one of the first bacterial avirulence factors to be identified [26,183], yet direct evidence for its enzymatic activity has been obtained only recently. It has long been known that AvrRpm1 facilitates the phosphorylation of RIN4 [25], but AvrRpm1 does not exhibit detectable kinase activity. Structural homology modeling identified a region of AvrRpm1 with similarity to a fold from the catalytic domain of poly(ADP-ribosyl)polymerase-1, and the mutation of putative catalytic residues abolished the function of AvrRpm1 in virulence or avirulence, depending on the genetic background of the host [184]. Furthermore, a recent experiment involving transient co-expression of AvrRpm1 and a soybean RIN4 homolog (GmRIN4b) in *N. benthamiana* followed by immunoprecipitation and mass spectrometry revealed two sites of mono-ADP-ribosylation in GmRIN4b [27]. This AvrRpm1-mediated modification was also detected in GmRIN4a, Arabidopsis RIN4, and several other NOI domain-containing proteins from Arabidopsis [27]. Amino acid substitutions in the putative catalytic residues of AvrRpm1 impaired this activity. In terms of functional relevance, RIN4 phosphorylation at threonine-166 is necessary and sufficient for activation of RPM1-mediated ETI [89], and the ADP-ribosylation of RIN4 at aspartic acid-153 promotes this phosphorylation, contributing additively to RPM1 activation [27]. On the virulence side, AvrRpm1-mediated ADP-ribosylation of RIN4 promotes the association of RIN4 with exocyst subunits EXO70B1, EXO70E1, EXO70E2, and EXO70F1, which is speculated to interfere with PAMP-induced callose deposition to compromise PTI [27]. A potential connection between EXO70 proteins and ADP-ribosylation of RIN4 by HopF2 is intriguing but remains to be explored.

While a relatively small number of examples of ADP-RT T3SEs are known, it is interesting to note that all of the currently known ADP-RT T3SEs catalyze mono-ADP-ribosylation. This includes XopAI from *Xanthomonas* *axonopodis* pv. *citri*, whose solved crystal structure resembles a mADP-RT but whose activity and substrate(s) remain to be demonstrated [185]. Endogenous mADP-RTs in prokaryotes are primarily known to regulate enzymes involved in nitrogen fixation [186], and it is possible that T3SEs could have evolved from these enzymes. Alternatively, genes encoding ADP-RTs could originate from lysogenic phages. Evidence for this mechanism comes from cholera toxin, whose two components are encoded by the genome of the bacteriophage CTXφ, which exists as a prophage integrated into the *Vibrio cholerae* genome [187,188]. A similar example of “lysogenic conversion” remains to be shown in phytopathogens, but the *P. syringae* and *Xanthomonas* spp. genomes contain prophage sequences, some of which harbor virulence factors [189,190,191]. Horizontal gene transfer from animal pathogens to plant pathogens is another avenue for T3SE evolution. Overall, ADP-RT T3SEs comprise a diverse group in sequence, structure, and substrates. There appears to be considerable variation in T3SE ADP-RT activity, with HopU1 exhibiting significantly greater activity than HopO1-1 [32]. The ADP-RT activity of HopF2 is quite weak and could only be demonstrated in vitro using biotin-labeled NAD [29], which is likely more sensitive than previous in vitro assays or in vivo radiolabeling assays that were unable to detect ADP-RT activity [28]. Direct in vitro comparisons with AvrRpm1 are complicated by the instability of the recombinant protein, such that AvrRpm1 ADP-RT activity has only been detected in planta thus far [27,184].

## 5. Acetyltransferase T3SEs

The YopJ/AvrRxv/HopZ family of T3SEs is an unusual T3SE family with acetyltransferase activity and is present in bacteria infecting mammals as well as plants. These T3SEs contain a conserved catalytic triad composed of histidine, glutamic acid, and cysteine and were originally believed to be cysteine proteases [192]. Well-characterized members of the superfamily are found in *P. syringae* (HopZ), *Xanthomonas* species (AvrRxv, AvrBsT, XopJ, and others), *R. solanacearum* (PopP2), and *Erwinia* species, as well as in animal-infecting bacterial genera such as *Yersinia* (YopJ), *Salmonella* (AvrA), and *Vibrio* (VopJ) [149]. Within *P. syringae*, HopZ1 diversified in response to recognition from the host immune system [150,193,194]. Other members of the HopZ family in *P. syringae* appear to have been acquired by horizontal gene transfer from *Xanthomonas* (HopZ2, HopZ4, and HopZ5) or *Erwinia* species (HopZ3) [46,150,195]. PopP2 is mostly closely related to a T3SE from *Xanthomonas* that is recognized in cabbage [150,196]. Here, we focus on members of the YopJ/AvrRxv/HopZ family that have acetyltransferase activity and which are found in plant pathogens [17,19,23,197]. Acetyltransferase activity is activated by inositol phosphate after the T3SE is introduced into the host cell [17,19,23,197,198]. 

### 5.1. T3SEs That Target the Cytoskeleton—HopZ1a and AvrBsT

HopZ1a and AvrBsT interfere with microtubule networks, which are important for vesicular trafficking and plant immunity. HopZ1a was found to interact with tubulin from human cells by LC-MS/MS and with tubulin from plant extracts by co-immunoprecipitation, as well as with polymerized tubulin through a microtubule sedimentation assay. HopZ1a acetylates tubulin in vitro, as detected by the incorporation of ^14^C-labeled acetyl CoA. This causes the disruption of microtubule networks and reduces trafficking to the apoplastic space [19]. As a result, PTI is suppressed, as observed by reduced callose deposition, reduced ROS production, and suppression of MAPK activation [19,199]. AvrBsT was shown to interact with microtubule-associated protein ACIP1 (ACETYLATED INTERACTING PROTEIN 1) in yeast and in GST pull-down assays. AvrBsT acetylates ACIP1 and promotes the formation of ACIP1 aggregates, disrupting the normal localization of this protein to rod-like microtubule structures [17]. Silencing of ACIP1 in Arabidopsis results in weaker PTI and ETI. Interestingly, AvrBsT does not acetylate tubulin and HopZ1a does not acetylate ACIP1, indicating that they have different strategies for disrupting microtubule networks. It is noteworthy that several other YopJ family members also appear to affect vesicular trafficking. XopJ interferes with the secretion of an apoplastic version of GFP, suggesting that it affects vesicular trafficking [49]. HopZ2 interacts with MLO2, which regulates vesicular trafficking dependent on the PEN1 syntaxin in response to pathogens [19,200,201]. Acetyltransferase activity has not been shown for XopJ or HopZ2, although the putative acetyltransferase catalytic cysteine is required for the virulence-promoting activity of both T3SEs [49,193].

### 5.2. T3SEs That Target Transcriptional Regulation—HopZ1a and PopP2

Some acetyltransferase T3SEs target transcription factors that regulate host susceptibility to infection. Similar to HopX1 (see Section 2.1), HopZ1a induces JA signaling by targeting JAZ proteins. As the SA and JA pathways are antagonistic, this results in lower SA production and reduced immune responses. HopZ1a was found to interact with soybean GmJAZ1 using yeast two-hybrid assays, in vitro pull-down assays, and bimolecular fluorescence complementation (BiFC) experiments [20]. This interaction depends on the Jas motif which is conserved within the JAZ protein family [202]. In addition, HopZ1a interacts with Arabidopsis JAZ2, JAZ5, JAZ6, JAZ8, and JAZ12 in vitro, and the JAZ6 interaction was confirmed in planta [202]. HopZ1a acetylates GmJAZ1 and AtJAZ6 proteins in vitro [20]. This results in the degradation of JAZ proteins and activation of JA signaling. Interestingly, some *P. syringae* strains carry the phytotoxin coronatine, which is a mimic of JA and causes JAZ degradation [147]. Thus *P. syringae* has multiple strategies to promote susceptibility through the JA pathway.

PopP2 targets the WRKY family of transcription factors, characterized by a highly conserved WRKYGQK motif within a DNA-binding domain that adopts a zinc finger structure [203]. The PopP2-mediated acetylation of multiple WRKY proteins can be detected using in planta and in vitro acetylation assays, and this modification interferes with DNA binding by WRKYs [24]. For WRKY22, the primary acetylation site was pinpointed to the C-terminal lysine residue of the WRKYGQK motif, which likely explains the consequent impairment of DNA binding [24]. While there are many WRKYs with roles in biotic stress, PopP2 appears to have some specificity for the WRKYs it acetylates [24,203]. Ultimately, the acetylation of WRKY transcription factors by PopP2 causes a dampening of PTI and associated immune-triggered transcriptional activation. 

### 5.3. T3SEs That Target Secondary Metabolites—HopZ1a and HopZ1b

HopZ1a and HopZ1b affect the production of isoflavones, which are secondary metabolites that influence plant–microbe interactions. Both T3SEs interact with a soybean 2-hydroxyisoflavanone dehydratase (GmHID1), as shown with yeast two-hybrid assays, in vitro pull-down experiments, and BiFC detection. GmHID1 is important for the synthesis of isoflavones such as daidzein and genistein [204]. When *P. syringae* infects soybean, *GmHID1* expression is induced and daidzein is produced [204]. HopZ1b suppresses daidzein production, while HopZ1a enhances it [204]. Silencing *GmHID1* results in higher growth of *P. syringae* [204]. While GmHID1 has not been shown to be acetylated by HopZ1b, the catalytic cysteine of HopZ1b is necessary for its virulence function.

### 5.4. T3SEs Whose Acetyltransferase Activity Triggers ETI—HopZ1a and PopP2

The acetylation activity of HopZ1a and PopP2 is critical for triggering immune responses [193,194]. HopZ1a is recognized by the pseudokinase HOPZ ETI-DEFICIENT 1 (ZED1) and the NLR ZAR1, which were both identified in genetic screens [21,194]. HopZ1a and ZED1 interact, as shown by yeast two-hybrid assays, BiFC, and surface plasmon resonance technology. HopZ1a acetylates ZED1 [21] as well as a related family of RCLKs known as PBS1-like kinases (PBLs), which form a ternary complex with ZED1 and ZAR1 [205]. ZED1 and ZAR1 normally interact in the absence of pathogens to prevent autoactivation of ZAR1 [21,206,207]. Acetylation of ZED1 is believed to cause conformational changes to ZAR1, which triggers ETI [21,206,207]. After activation, ZAR1 likely forms a plasma membrane-associated pore which aids in cell death [208,209,210]. HopZ5 is also an acetyltransferase whose activity is necessary for resistance; however, the targets of its activity are unknown [211]. XopJ4 is recognized in *N. benthamiana* through ZAR1 and a homolog of ZED1, JIM2 (XOPJ4 IMMUNITY 2); however, enzymatic activity has not been demonstrated for XopJ4 [212].

PopP2 is recognized by RRS1 (RESISTANT TO RALSTONIA SOLANACEARUM 1), a NLR with an integrated WRKY domain, in complex with the NLR RPS4 (RESISTANT TO P. SYRINGAE 4) [23,213,214]. RRS1 was first identified in Arabidopsis ecotypes that differ in their resistance to *R. solanacearum* carrying PopP2 [213,214]. Subsequently, RRS1 was shown to function with RPS4 to trigger ETI [215,216]. PopP2 acetylates RRS1 in the WRKY domain, which disrupts its association with DNA and activates RPS4-mediated ETI [24]. The WRKY domain of RRS1 thus acts as an integrated decoy domain for PopP2 recognition. 

### 5.5. T3SEs That Suppress the HR—HopZ3 and AvrBsT

HopZ3 contributes to the epiphytic growth of *P. syringae* pv. *syringae* B728a by suppressing the HR associated with multiple effectors, including AvrB3 and AvrRpm1, which are recognized by RPM1 (see Section 2) [22,217,218]. HopZ3 interacts with AvrB3 and AvrRpm1, as well as RIN4 and several RLCKs, including RIPK, with evidence from yeast two-hybrid assays, BiFC experiments, and GST pull-downs [22]. While HopZ3 is localized to the nucleus when expressed alone [193,199], its interaction with AvrB3, AvrRpm1, RIN4, or RIPK relocalizes it to the cell periphery, in the vicinity of the RPM1 immune complex [22]. HopZ3 acetylates AvrB3 and AvrRpm1, as well as the plant RLCKs RIN4, RIPK, PBS1, and BIK1 [22]. HopZ3 exhibits varying activity on different RLCK ligands because it does not acetylate MPK4 even though it can interact with MPK4. Interestingly, HopZ3 acetylates RIPK and AvrB3 in regions that are important for kinase activity, protein–protein interactions, and/or AvrB3-triggered immunity, thereby blocking plant immunity [22]. HopZ3 reduces AvrB3/AvrRpm1-mediated phosphorylation of RIN4, which normally triggers immunity through RPM1 [22]. In addition, transgenic HopZ3-expressing Arabidopsis lines suppress PTI, as well as flg22-triggered ROS production and MAPK activation, in a catalytic cysteine-dependent manner [199]. Thus, HopZ3 has multiple strategies for immune suppression.

AvrBsT suppresses the HR that is induced by an unrelated *Xanthomonas* T3SE, AvrBs1 [18]. This suppression depends on the catalytic triad of AvrBsT and involves SNF1-RELATED KINASE 1 (SnRK1) [18]. While AvrBsT has been shown to possess acetyltransferase activity [17], acetylation of SnRK1 has not yet been shown [18].

## 6. T3SEs That Alter Host Protein Phosphorylation

A wide range of proteins in both prokaryotes and eukaryotes are regulated by reversible phosphorylation [219,220], including proteins at the molecular interface of host–microbe interactions. Phosphorylation is particularly important for immune signaling following PAMP perception, including auto- and transphosphorylation events within RLK–RLCK complexes prior to the activation of downstream kinase signaling cascades [4,221]. The elicitation and execution of ETI responses are also influenced by phosphorylation [12]. As such, T3SEs have evolved to manipulate various members of the defensive phosphoproteome.

### 6.1. T3SEs Targeting PRRs—HopAO1

One efficient mechanism for T3SE-mediated suppression of PTI involves disabling the PRRs responsible for PAMP perception. Sequence analysis of the *P. syringae* T3SE HopAO1 indicated the presence of a conserved [LIVMF]HCxAGxxR[STC][STAG] motif found in the catalytic site of protein tyrosine phosphatase enzymes [222,223,224]. Enzymatic activity was demonstrated in vitro using an artificial substrate (paranitrophenyl phosphate) as well as phosphotyrosine peptides derived from the insulin receptor or the epidermal growth factor receptor [223,224]. In Arabidopsis, transgenic expression of HopAO1 enhances the growth of a type III secretion-deficient *P. syringae* pv. *tomato* DC3000 *hrpA*^−^ mutant and suppresses callose deposition elicited by this strain in a catalytic site-dependent manner, suggesting that HopAO1 targets PTI [225]. Indeed, HopAO1 interacts with the kinase domains of EFR and FLS2 and reduces EFR autophosphorylation [61]. This reduction is only partially dependent on HopAO1 catalytic activity, suggesting that the physical interaction with HopAO1 also contributes to EFR inhibition [61]. In this system, FLS2 kinase activity was too weak to assess the impact of HopAO1, but the interaction of HopAO1 with FLS2 and the suppression of flg22-induced responses by this T3SE suggest that FLS2 is affected in a similar manner. More recently, a G-type lectin RLK designated LIPOOLIGOSACCHARIDE-SPECIFIC REDUCED ELICITATION (LORE) was found to perceive medium-chain 3-hydroxy fatty acids as PAMPs from *Pseudomonas* spp. and autophosphorylate prior to PTI activation [62,226]. As with EFR, HopAO1 interacts with LORE and reduces its autophosphorylation as a mechanism of PTI suppression [62].

### 6.2. T3SEs Targeting MAPK Signaling Cascades—HopAI1 and XopAU

Downstream of PRRs, MAPK signaling cascades are also targeted by T3SEs. The T3SE HopAI1 from *P. syringae* can suppress a range of PTI responses, including flg22-induced marker gene expression, callose deposition, ROS burst, and activation of the MAPKs MPK3 and MPK6 [59,227]. In vitro pull-down experiments and in planta co-IP assays indicated that HopAI1 interacts with MPK3/6, and phospho-specific antibodies were used to demonstrate that HopAI1 dephosphorylates MPK3/6 [59]. MAPKs are phosphorylated at TXY motifs on both threonine and tyrosine residues [228], but mass spectrometry analysis showed that only the threonine is dephosphorylated, indicating that HopAI1 is a phosphothreonine lyase [59]. Examination of a homologous T3SE (OspF) from *Shigella flexneri* [229] identified a catalytic histidine residue that was shown to be critical for HopAI1-mediated MAPK dephosphorylation and PTI suppression [59]. Mechanistically, HopAI1 and OspF likely act by attacking the C-O bond of phosphorylated threonine, yielding a dephosphorylated residue that lacks a hydroxyl group and thus cannot be rephosphorylated, which differs from other phosphatases. Interestingly, HopAI1 activity can be recognized in hosts that possess the NLR SUPPRESSOR OF MKK1 MKK2 2 (SUMM2) [60]. A key component of this recognition is the MAPK MPK4, which also interacts with HopAI1 and whose flg22-induced kinase activity is impaired by HopAI1, although direct dephosphorylation was not tested. To detect HopAI1 activity, SUMM2 interacts with CALMODULIN-BINDING RECEPTOR-LIKE CYTOPLASMIC KINASE 3 (CRCK3), which in turn interacts with and is phosphorylated by MPK4 in vivo [230]. It is likely that CRCK3 acts as a decoy, and that ETI is stimulated when CRCK3 steady-state phosphorylation levels are reduced by HopAI1-mediated disruption of MPK4 activity. Interestingly, HopAI1 activity can be mitigated by host-mediated S-nitrosylation of HopAI1 at a conserved cysteine residue, which reduces the ability of HopAI1 to suppress MAPK activity [231].

In contrast to phosphatase-mediated suppression of MAPK signaling, the *X. euvesicatoria* T3SE XopAU stimulates this signaling cascade. A search of the XopAU sequence for conserved domains predicted that this T3SE belongs to the serine/threonine kinase family [58]. This was verified in vitro by the detection of autophosphorylation in the presence of radiolabeled ATP, and was shown to be dependent on a conserved ATP binding site (lysine-240). Transient expression of XopAU in pepper and *N. benthamiana* causes cell death, MAPK phosphorylation, and PR protein expression, all of which are lost in a kinase-deficient K240A mutant [58]. Yeast two-hybrid analyses revealed that XopAU interacts with MAP kinase kinases, including tomato MKK2, *N. benthamiana* MEK2, and pepper MKK2 [58]. In vitro and in planta assays confirmed that XopAU phosphorylates tomato MKK2. Given that the phosphorylation and subsequent activation of MKK2 result in host cell death and immune activation, the virulence function of XopAU remains unclear.

### 6.3. T3SEs That Modulate ETI by Phosphorylation—HopBF1 and AvrRxo1

Effector-triggered immunity can also be modulated by phosphorylation. The T3SE HopBF1 is widely distributed across bacterial genera, including plant and animal pathogens [57]. Bioinformatic analyses suggested that HopBF1 is distantly related to classical protein kinases and aminoglycoside phosphotransferases. The crystal structure of HopBF1 from the human pathogen *Ewiginella americana* revealed that the T3SE includes a minimal and unusual protein kinase-like fold. Functionally, HopBF1 interacts with and phosphorylates the chaperone Hsp90 from both plant and human sources [57]. This phosphorylation reduces the ATPase activity used by Hsp90 to catalyze the folding of client proteins [57]. While the impact of HopBF1 on Hsp90 chaperone function has primarily been evaluated in mammalian cells, HopBF1 was shown to delay and generally reduce the HR elicited by an autoactive variant of the NLR RPM1 in *N. benthamiana* [57]. As such, the virulence function of HopBF1 is thought to derive from the inhibition of Hsp90 chaperone activity, which destabilizes NLRs and dampens ETI. In addition, HopBF1 actually promotes cell death in the later stages of compatible pathogen interactions, suggesting a second activity in facilitating the transition of *P. syringae* to a necrotrophic lifestyle in advanced infections [57].

Cell death promotion is also exhibited by the T3SE AvrRxo1, which is found in a wide range of *Xanthomonas*, *Burkholderia*, and *Acidovorax* species [232]. The crystal structure of AvrRxo1 resembles the N-terminal kinase domain of T4 polynucleotide kinase from an enterobacterial phage [233]. A putative ATP-binding residue (threonine-167) is required for AvrRxo1-mediated cell death in *Escherichia coli* as well as in several different plant species [233]. In rice, however, AvrRxo1 enhances the virulence of *X. oryzae* pv. *oryzicola*. Biochemical analyses demonstrated that AvrRxo1 phosphorylates NAD and its precursor NAAD at the 3′ hydroxy of their adenosines to form 3′-NADP and 3′-NAADP, respectively [54,55]. These products may interfere with the function of NADP-dependent enzymes, and 3′-NAADP may modulate Ca^2+^ signaling to promote bacterial virulence. A recent investigation of drought-induced immunity found that AvrRxo1 also interacts with the dehydration-induced cysteine protease RESPONSIVE TO DESICCATION 21 (RD21A) and the E3 ubiquitin ligase SEVEN IN ABSENTIA 4 (SINAT4) [56]. Here, AvrRxo1 significantly enhances SINAT4 self-ubiquitination, and SINAT4 promotes RD21A degradation via the 26S proteasome pathway to compromise drought-induced immunity [56]. Although direct phosphorylation of SINAT4 by AvrRxo1 was not tested, the activity of AvrRxo1 in this context was dependent on the ATP-binding residue threonine-167. 

## 7. T3SEs with Other Activities

A number of T3SEs have evolved unique activities that illustrate the utility of deploying a biochemically diverse collection of T3SEs to manipulate an equally diverse range of host targets. Here, we highlight some of these unique T3SEs and their characterized virulence functions.

### 7.1. T3SEs with Nucleoside Hydrolase Activity—XopQ and HopQ1

The modification of endogenous small molecules in host cells can significantly impact plant immunity. The amino acid sequences of XopQ and HopQ1 bear some resemblance to inosine/uridine-preferring nucleoside hydrolase enzymes, including a conserved DXXXDXDD nucleoside binding motif [85,86,234]. Nucleoside hydrolases (NHs) cleave the N-glycosidic bond of nucleosides to release a ribose sugar from the nucleobase. Indeed, the crystal structure of XopQ from *X. oryzae* pv. *oryzae* was deduced from this protein in complex with adenosine diphosphate ribose, which contains a ribosyl moiety and serves as an important regulator of Ca^2+^-mediated immune signaling [235]. Furthermore, the virulence functions of both XopQ and HopQ1 require an intact nucleoside binding site [85,234]. For XopQ, NH activity could not be demonstrated in vitro using common plant nucleosides, but weak ribose hydrolase activity was detected with the substrate p-nitrophenyl β-D-ribofuranoside [86]. A XopQ nucleoside binding site mutant displayed lower substrate binding affinity and cleavage efficiency. As such, XopQ is likely a functional NH whose natural substrate remains to be confirmed. In *N. benthamiana*, XopQ triggers ETI following its detection by the NLR RECOGNITION OF XOPQ 1 (ROQ1) [120,236]. Interestingly, this recognition involves a direct interaction between the C-terminus of ROQ1 and the putative NH active site of XopQ, serving to block XopQ ligand binding in addition to eliciting an HR.

The biochemical activity of HopQ1 is more thoroughly understood. Transgenic expression of wild-type HopQ1, but not a nucleoside binding site mutant, suppresses the ROS burst and MPK3/MPK6 phosphorylation that are induced by flg22 treatment and inhibits expression of the *FLS2* gene [85]. Intriguingly, HopQ1 expression in seedlings caused a number of developmental defects reminiscent of cytokinin signaling mutants, also in a nucleoside binding site-dependent manner. This observation spurred additional phytochemical analyses, revealing that HopQ1 expression stimulates the production of various cytokinins, including N6-(2-isopentenyl)adenine (iP), trans-zeatin, cis-zeatin, and dihydrozeatin, as well as the upregulation of cytokinin-responsive genes [85]. Exogenous trans-zeatin mimics HopQ1 expression phenotypes in terms of reduced *FLS2* expression and attenuated PTI responses [85]. In vitro, HopQ1 was shown to hydrolyze the iP precursor, iP-9-riboside-5’-monophosphate, in a nucleoside binding site-dependent manner [85]. Overall, it appears that the NH activity of HopQ1 facilitates cytokinin accumulation to suppress PTI. It is possible that the virulence function of XopQ involves a similar mechanism.

### 7.2. Uridylyltransferase—AvrAC

Rather than directly altering the phosphostatus of in planta targets, some T3SEs have instead evolved to block the activity of host kinases. The T3SE AvrAC from *Xanthomonas campestris* pv. *campestris* inhibits plant immunity through the targeting of the receptor-like kinases BIK1 and RIPK, which are known to play roles in PTI and ETI, respectively [79,90,237]. AvrAC is composed of three domains: an N-terminal domain of unknown function, a leucine-rich repeat (LRR) domain, and a C-terminal region with a Fic (filamentation induced by cAMP) domain that includes a conserved HPFx(D/E)GN(G/K)R motif necessary for nucleotide binding and catalysis [79,238]. Mass spectrometry analysis revealed multiple serine and threonine residues on AvrAC which possess mono-UMP (uridine 5′-monophosphate) modifications, indicating that AvrAC likely acts as a uridylyltransferase [79]. In vitro assays with radiolabeled UTP showed that BIK1 and RIPK are uridylylated upon incubation with AvrAC. Further, this uridylylation occurs specifically at conserved residues in the activation loop of these kinases, as AvrAC failed to uridylylate the BIK1(S236A) and BIK1(T237A) mutants [79]. UMP-modified BIK1 and RIPK exhibited reduced autophosphorylation in *E. coli*, indicating the uridylylation of conserved phosphorylation sites in the activation loops of both proteins prevents their phosphorylation and inhibits downstream signal transduction.

The modification of BIK1 and RIPK by AvrAC markedly compromises both PTI and ETI in Arabidopsis. Transgenic lines expressing *avrAC* supported significantly more growth of the type III secretion-deficient strains *X. campestris* pv. *campestris ΔhrcV* and *P. syringae* pv. *tomato ΔhrcC* [79]. Additionally, flg22-triggered MAPK activation and H_2_O_2_ accumulation were strongly diminished in the transgenic lines when compared to the wild type [79]. The *avrAC* transgenic lines also support significantly more growth of *P. syringae* pv. *tomato* strains delivering AvrB, which triggers RPM1-dependent ETI in Arabidopsis [79]. Consistent with this, plants pretreated with *X. campestris* pv. *campestris* do not exhibit an HR when subsequently infiltrated with *P. syringae* pv. *tomato* carrying *avrB*, whereas an HR was still observed in plants pretreated with *X. campestris* pv. *campestris ΔavrAC* and those receiving no pretreatment [79].

Notably, AvrAC plays a dual role in plant immunity, not only contributing to bacterial virulence but also triggering immunity in some plants. AvrAC was initially identified as an avirulence factor in Arabidopsis, as an *X. campestris* pv. *campestris* knockout of AvrAC was found to be more capable of causing disease than the wild-type strain when infiltrated into the Arabidopsis vasculature [239]. More recently, it was discovered that PBL2, a paralog of BIK1, is also uridylylated by AvrAC in planta [80]. The modification of PBL2 does not contribute to virulence but instead triggers immunity via recognition by the ZAR1–RKS1 complex [80,208,209]. These observations suggest that PBL2 may have evolved as a decoy to enable AvrAC detection.

### 7.3. Phytase—XopH

The T3SE XopH (also known as avrBs1.1) from *X. euvesicatoria* was initially believed to be a phosphatase due to its sequence similarity to known phosphatases [240,241]. While XopH does possess the conserved P loop and WPD loop residues associated with protein phosphatases, it demonstrates weaker enzymatic activity than other known phosphatases [81]. Instead, XopH appears to act primarily as a phytase. Phytases dephosphorylate phytate (*myo*-inositol *hexakis*phosphate or InsP_6_) to lower phosphorylated *myo-*inositol derivatives. In in vitro assays, XopH is capable of dephosphorylating phytate at position 1 to produce InsP_5_ [81]. Interestingly, XopH does not dephosphorylate alternative phytase substrates, indicating that it has high substrate specificity [81]. The phytase activity of XopH was also confirmed in planta, as *N. benthamiana* plants expressing *xopH* showed an accumulation of InsP_5_ concomitant with decreased InsP_6_ [81]. However, it remains unclear how this phytase activity contributes to virulence. When delivered by a *P. syringae* strain lacking the T3SE-rich conserved effector locus (CEL), XopH was shown to significantly inhibit callose deposition and enhance *P. syringae* pv. *tomato* DC3000 ΔCEL disease symptoms in Arabidopsis [242]. Additionally, XopH induces the expression of genes associated with ethylene and JA signaling pathways in *N. benthamiana* [81]. While the precise role of phytate and its derivatives in these effects is unclear, upregulation of JA/ethylene signaling could enhance host susceptibility to hemibiotrophic pathogens such as *X. euvesicatoria*. Additional virulence functions for XopH may include the liberation of phosphate for pathogen nutrition and/or the attenuation of host immune responses due to reduced InsP_6_ levels [243]. On the other hand, XopH is recognized in pepper by the protein Bs7 to trigger an HR [241]. This recognition requires an active phytase domain, suggesting Bs7 recognizes the biochemical activity of XopH rather than the physical protein [81].

### 7.4. Trehalose-6-Phosphate Synthase—RipTPS

The T3SE RipTPS from *R. solanacearum* is a conserved effector in the *R. solanacearum* complex that functions as a trehalose-6-phosphate synthase (TPS) within the trehalose biosynthetic pathway [82]. In plants, trehalose influences a range of processes, including plant growth and development, carbohydrate metabolism, and responses to abiotic stress [244]. This molecule has also been shown to promote pathogen virulence in multiple pathosystems, although the underlying mechanisms remain unclear [245]. Deletion of a trehalose synthase gene in the fungal pathogen *Magnaporthae oryzae* weakened its overall pathogenicity [246]. *Pseudomonas aeruginosa*, a pathogen often found in immunocompromised humans, can also cause soft rot symptoms in susceptible Arabidopsis ecotypes when infiltrated at high inoculum levels [247]. Similar to *M. oryzae*, bacterial growth and the severity of chlorosis on Arabidopsis infected with *P. aeruginosa* were dependent on trehalose synthesis [247]. While trehalose synthesis appears to contribute significantly to the virulence of plant pathogens, RipTPS remains the only known example of a TPS that is translocated into plant cells by the T3SS [82]. The enzymatic activity of this T3SE has been demonstrated by the ability of RipTPS to catalyze trehalose-6-phosphate biosynthesis in yeast and to rescue the growth defects of yeast TPS-deficient mutants, with both phenotypes being dependent on three putative catalytic residues: tyrosine-154, tryptophan-163, and aspartic acid-208 [82]. However, the contribution of RipTPS to *R. solanacearum* virulence is not known. *RipTPS* knockouts do not demonstrate any deficiencies in plant infection [82]. Given the known role of trehalose synthesis in promoting pathogen virulence, it is possible that RipTPS acts redundantly with another effector within the *R. solanacearum* T3SE suite. Conversely, RipTPS can activate plant immunity, as heterologous expression in *Nicotiana tabacum* elicits an HR [82]. Although trehalose has been previously implicated as an elicitor of plant defense, the HR in *N. tabacum* is triggered independently of RipTPS enzymatic activity [82,248]. RipTPS is currently the only T3SE known to play a role in trehalose synthesis, but RipTPS homologs do exist in other species, indicating that this T3SE activity may be more broadly deployed than previously thought [82].

### 7.5. Nudix Hydrolase—RipN

The *R. solanacearum* T3SE RipN was recently discovered to be a functional Nudix hydrolase [84]. Nudix hydrolases act on substrates composed of a nucleoside diphosphate linked to another moiety (x) and share a conserved Nudix motif of Gx5Ex5[UA]xREx2EExGU, where U is an aliphatic hydrophobic residue. Several Nudix substrates have been shown to play key roles as regulatory molecules in immune signaling. For example, the Arabidopsis Nudix hydrolase NUDT7 acts on ADP-ribose and NADH to regulate cellular antioxidant status and modulate plant defense responses [249]. Similarly, RipN has ADP-ribose/NADH pyrophosphorylase activity in vitro [84]. However, RipN does not appear to impact the levels of mono-/poly-ADP-ribosylated proteins in planta. Instead, RipN primarily affects NADH/NAD^+^ levels in planta, indicating that NADH is the preferred substrate for RipN [84]. Importantly, RipN Nudix hydrolase activity is necessary for its contribution to virulence. *RipN* transgenic Arabidopsis plants supported significantly higher populations of *P. syringae* and *R. solanacearum* compared to wild-type Arabidopsis or transgenic plants expressing a catalytically inactive *ripN* mutant [84]. Further, *RipN* transgenic plants demonstrated significantly decreased callose deposition when induced by the PAMP flg22 [84]. Taken together, these data suggest that RipN utilizes its Nudix hydrolase activity to modulate the levels of NADH/NAD^+^ in planta and ultimately suppress PTI responses.

### 7.6. γ-Glutamyl Cyclotransferase—RipAY

The *R. solanacearum* T3SE repertoire also includes RipAY, which interferes with redox homeostasis in the plant host. RipAY possesses a ChaC domain, which had previously been reported to be associated with γ-glutamyl cyclotransferase activity, specifically glutathione degradation in yeast and mammals [250]. Heterologous expression of RipAY in yeast resulted in inhibited growth and significantly decreased intracellular glutathione levels, confirming that RipAY also exhibits γ-glutamyl cyclotransferase activity to degrade glutathione [83]. Glutathione acts a master redox buffer in eukaryotes and is known to influence plant immune responses [251,252]. Inoculation of wild-type *R. solanacearum* into eggplant leaves resulted in a significant decrease in glutathione levels [83]. However, an *R. solanacearum ΔripAY* knockout was unable to alter glutathione accumulation, indicating that RipAY functions as a γ-glutamyl cyclotransferase in planta [83]. In competitive assays, RipAY significantly contributes to *R. solanacearum* fitness in eggplant, which implies a role for the manipulation of glutathione levels in promoting virulence [253]. In support of this, transient expression of RipAY in *N. benthamiana* resulted in a significant reduction in the flg22-triggered ROS burst and the SA-induced expression of the *N. benthamiana* ortholog of Arabidopsis *PR1*, *NbPR1* [254]. Arabidopsis plants deficient in glutathione accumulation similarly exhibit a reduced flg22-induced ROS burst [254]. In total, these data suggest that RipAY suppresses immune responses and that this suppression is dependent on its γ-glutamyl cyclotransferase activity to decrease glutathione levels. Notably, RipAY is activated in planta by host cytosolic thioredoxins to degrade glutathione specifically in eukaryotic cells [83,255,256,257]. Effector activation in planta is further discussed in Section 10 “Context Matters”.

## 8. T3SEs with Indirect and/or Non-Enzymatic Mechanisms of Action

While we have primarily focused on characterized T3SEs that catalyze a specific biochemical reaction, several other effectors contribute to pathogen virulence independently of any direct enzymatic activity. The interaction of these T3SEs with host proteins may allosterically inhibit host enzymes, occlude substrate binding sites, disrupt oligomeric structures, sequester proteins away from their intended interactors, or recruit enzymes to act on a given target protein. Other T3SEs bind DNA to manipulate host gene expression.

### 8.1. T3SEs That Target PRRs and RLCKs—AvrPto

PRRs and RLCKs are commonly targeted by T3SEs, as described in multiple previous sections. AvrPto interferes with PRR signaling by physically binding the kinase domain of PRRs, including FLS2, EFR, CERK1, and possibly BAK1/SERK3 [65,95,96]. Physical occlusion of the kinase domain by AvrPto prevents signaling through PRRs after recognition of PAMPs and suppresses downstream immune responses [237,258]. AvrPto can also interact with a number of other kinases, including the brassinosteroid-signaling kinases BSK1 and BSK3 [95,96]. Consistent with this, transgenic expression of AvrPto in Arabidopsis appears to result in phenotypes that resemble brassinosteroid-insensitive mutants [65]. Interestingly, the targeting of kinases by AvrPto is co-opted by the host for recognition. AvrPto is recognized by an oligomeric complex composed of the RLCK Pto and NLR Prf [97,259,260,261,262]. AvrPto is able to inhibit the kinase activity of Pto [263]. However, since there are multiple Pto molecules in the Pto/Prf complex, inhibition of the kinase activity of one Pto molecule disrupts the interaction between Pto and Prf, which then activates the kinase activity of another Pto molecule [264]. This results in transphosphorylation of other Pto proteins in the Pto/Prf complex and induces ETI [264].

In addition, AvrPto is able to suppress cell death induced by the overexpression of SUPPRESSOR OF BAK1-INTERACTING RECEPTOR-LIKE KINASE 1 (SOBIR1) in *N. benthamiana*, as well as cell death induced by a fungal effector, Avr4, in *N. benthamiana* plants that express tomato RLP Cf-4 (*Cladosporium fulvum*-4) [98]. Cf-4 functions with SOBIR1 to recognize Avr4, a secreted protein from the fungal pathogen *Cladosporium fulvum*. While AvrPto interacts with SOBIR1 and the RLK SERK3, it does not affect Cf-4/SlSOBIR1/SlSERK3 complex formation [98]. It is not yet clear whether AvrPto interferes with SOBIR1/SERK3 phosphorylation status or whether it blocks the kinase domain of these proteins as seen with other virulence targets [98].

### 8.2. T3SEs That Target 14-3-3 Proteins—XopN, HopQ, XopQ, XopX, AvrRxv, XopE1, XopE2, and XopO

It is increasingly apparent that 14-3-3 proteins are common T3SE targets, especially for *Xanthomonas* pathogens. As a group, 14-3-3 proteins comprise a eukaryote-specific protein family that regulates signaling pathways by sensing the phosphorylation status of client proteins and then modulating client activity [265]. Several members of this family are known to influence plant immunity. One of the most well-characterized 14-3-3 protein-targeting T3SEs is XopN, which suppresses PTI responses in rice and tomato and is required for full virulence in various *Xanthomonas* species [266,267,268]. Yeast two-hybrid screens and other interaction assays demonstrated that XopN interacts with the protein TOMATO FOURTEEN-THREE-THREE 1 (TFT1) as well as the cytosolic domain of an LRR-RLK designated TOMATO ATYPICAL RECEPTOR-LIKE KINASE 1 (TARK1) [267]. Both TFT1 and TARK1 are positive regulators of immunity, although TARK1 lacks autophosphorylation activity and is likely a pseudokinase [117,267]. Interestingly, TFT1 and TARK1 interact in the presence of XopN, and it is thought that XopN promotes pathogen virulence by acting as a scaffold to stabilize TFT1–TARK1 interactions and sequester TFT1 in a non-functional PTI signaling complex [117]. The *P. syringae* T3SE HopQ also suppresses PTI through interactions with the 14-3-3 proteins TFT1 and TFT5 in tomato [110]. The underlying mechanisms for this suppression remain to be elucidated, but the 14-3-3 proteins exhibit a nucleocytoplasmic distribution when transiently expressed in *N. benthamiana*, yet are mostly cytoplasmic when co-expressed with HopQ1, so altered subcellular localization may be part of the virulence function of HopQ1 [269]. Similarly, PTI is suppressed in rice by the interaction of XopQ from *X. oryzae* pv. *oryzae* and the rice 14-3-3 proteins Gf14f and Gf14g [119], as well as by XopX interacting with Gf14d and Gf14e [121]. In *X. euvesicatoria*, XopQ was shown to suppress ETI in tomato, pepper, and *N. benthamiana* [270]. This suppression is dependent on the interaction of XopQ with the tomato 14-3-3 protein TFT4 and its homologs in other solanaceous species. In this context, XopQ may inhibit the function of TFT4 as a positive regulator of ETI or block interactions with other ETI-promoting proteins. In contrast, AvrRxv (*X. euvesicatoria*) interacts with a tomato 14-3-3 protein designated AVRRXV INTERACTOR 1 (ARI1), which may enhance AvrRxv-dependent ETI [102]. Finally, a systematic analysis of *X. euvesicatoria* T3SEs revealed that XopE1, XopE2, and XopO interact with multiple 14-3-3 proteins from tomato, and in planta assays suggested that these T3SEs may function redundantly to reduce the development of chlorotic symptoms in tomato leaves during infection [271].

### 8.3. T3SEs That Target the Cytoskeleton—HopW1, HopG1, and HopE1

The cytoskeleton represents another venue where interactions with T3SE proteins can alter plant immunity. The *P. syringae* T3SE HopW1 interacts directly with actin, causing a reduction in dynamic actin filaments (F-actin) and an overall disruption of the actin cytoskeleton [111]. Consequently, both endocytosis and intracellular protein trafficking are impaired and host susceptibility to infection is enhanced. The actin cytoskeleton is also targeted by HopG1 (*P. syringae*), although this T3SE is specifically localized to mitochondria [105,106]. Here, HopG1 interacts with an actin-associated kinesin motor protein and not actin itself [106,272]. Immunoprecipitation experiments suggest that HopG1 does not disrupt the interaction between kinesin and actin but rather inhibits kinesin function through a yet-undetermined mechanism [106]. Ultimately, HopG1 causes actin filament bundling and an impairment of mitochondrial respiration, which are associated with PTI suppression and increased chlorotic symptom development following inoculation with a virulent strain of *P. syringae* [105,106]. The *P. syringae* T3SE HopE1 also suppresses PTI, and yeast two-hybrid screens revealed that HopE1 interacts with calmodulin and the microtubule-associated protein MAP65-1 [104]. Furthermore, calmodulin and MAP65-1 also interact, but only when HopE1 is present [104]. These interactions cause MAP65-1 to dissociate from microtubules, which impairs protein secretion, including the release of the pathogenesis-related protein PR-1 into the extracellular fluid of SA-treated plants [104]. Overall, then, HopE1 co-opts calmodulin as a co-factor to bind MAP65-1 and sequester it away from the microtubule network. Notably, HopZ1a and AvrBsT also target microtubules or microtubule-associated proteins to disrupt immune responses (see Section 5).

### 8.4. T3SEs That Manipulate Oligomeric Complexes—RipAC and AvrBsT

The strategy of manipulating host protein complexes is also illustrated by the T3SE RipAC from *R. solanacearum*. This effector inhibits ETI but not cell death caused by general elicitors such as the *Phytophthora infestans* secreted protein INF1 or the mammalian pro-apoptotic protein Bax, indicating specificity for programmed cell death responses arising from T3SE recognition [112,113]. Interestingly, RipAC contains tandem LRR motifs that are required for ETI suppression, suggesting that it may act as a scaffold for protein–protein interactions. Indeed, two independent studies found that RipAC interacts with homologs of SUPPRESSOR OF G2 ALLELE OF *skp1* (SGT1), a positive regulator of plant immunity [112,113]. A molecular chaperone complex comprising SGT1, RAR1, and Hsp90 is required for NLR stability and function [273], and RipAC interferes with the SGT1–RAR1 interaction in an LRR-dependent manner [113]. This T3SE also blocks the association of SGT1 with the MAPKs MPK3 and MPK6, thus inhibiting the phosphorylation of SGT1 that normally follows ETI activation [112]. The consequent impairment of NLR function underlies the attenuation of ETI by RipAC.

On the other hand, ETI is elicited following the manipulation of a SGT1-containing complex by the *X. euvesicatoria* T3SE AvrBsT. The RLCK PATHOGEN-INDUCED PROTEIN KINASE 1 (PIK1) normally interacts with and phosphorylates SGT1, likely as a positive regulator of basal defenses [92]. AvrBsT also binds SGT1, forming an AvrBsT–SGT1–SGT1–PIK1 complex in which AvrBsT is phosphorylated instead of SGT1 prior to PIK1 dissociation from the complex. The resulting AvrBsT–SGT1–SGT1 association is recognized by a yet-unidentified NLR to stimulate ETI. This avirulence function is independent of the acetyltransferase activity of AvrBsT and appears to derive from the physical occlusion of SGT1 phosphorylation sites. 

### 8.5. T3SEs That Modulate the Ubiquitin–Proteasome System—RipAC, XopP, AvrRps4, HopBB1, and HopM1 

While some T3SEs mimic enzymatic components of the UPS (see Section 3), others manipulate the UPS via non-enzymatic mechanisms. In addition to targeting SGT1 complexes, RipAC also directly interacts with the E3 ubiquitin ligase PUB4 in both Arabidopsis and tomato [114]. PUB4 regulates BIK1 homeostasis by preventing BIK1 accumulation prior to PAMP perception and increasing its accumulation following elicitation of PTI [114]. BIK1 plays a key role in immunity as it is involved in several PTI responses, including ROS production, calcium signaling, callose deposition, and stomatal closure [237,274,275]. RipAC association with PUB4 results in overrepresentation of the pre-elicitation form of PUB4, leading to a decrease in BIK1 accumulation and an attenuation of these PTI responses [114]. Similar to RipAC, the *X. oryzae* pv. *oryzae* T3SE XopP suppresses the activity of a host E3 ubiquitin ligase, OsPUB44 [118]. OsPUB44 is a positive regulator of PTI in rice, as chitin-triggered expression of PTI reporter genes is reduced in *OsPUB44* RNAi-silenced lines [118]. XopP physically binds the U-box domain of OsPUB44 and reduces its ligase activity [118]. Though direct targets have not yet been identified, it is possible that OsPUB44 serves a role in regulating the homeostasis of PTI signaling components similar to PUB4 in Arabidopsis.

Along the same lines, the virulence function of the *P. syringae* T3SE AvrRps4 was recently shown to involve an interaction with an iron-sensing E3 ubiquitin ligase designated BRUTUS (BTS) [99]. Iron deficiency stabilizes the BTS protein, which mediates the 26S proteasome-dependent degradation of transcription factors such as BASIC HELIX-LOOP-HELIX 115 (bHLH115) and IAA-LEUCINE RESISTANT 3 (ILR3), thus modulating iron-responsive gene expression [276]. AvrRps4 directly interacts with and suppresses the E3 ligase activity of BTS to upregulate bHLH115/ILR3-dependent gene expression and increase the concentration of iron in the apoplast of Arabidopsis leaves [99]. This metabolic reprogramming directly enhances in planta bacterial growth, as also shown with Arabidopsis mutants exhibiting increased iron accumulation or plants treated with exogenous iron, which all support higher populations of *P. syringae* [99]. The impact of AvrRps4 on pathogen growth is dependent on the genetic background of the host because AvrRps4 is recognized by the NLR pair RRS1/RPS4 [24,100]. As with PopP2, AvrRps4 is detected by the integrated WRKY domain of RRS1, and the lack of demonstrated enzymatic activity for AvrRps4 suggests that recognition is mediated purely by the physical WRKY–AvrRps4 interaction. Notably, RRS1/RPS4-mediated recognition of AvrRps4 blocks the iron accumulation phenotype, although the underlying mechanism is not yet clear [99]. In terms of additional virulence targets, AvrRps4 interacts with several WRKY proteins as well as with EDS1 within the RRS1/RPS4 complex, but the specific contribution of these interactions to the promotion of pathogen virulence remains to be conclusively demonstrated [100,277]. 

Yet another virulence strategy involves promoting the activity of the host UPS to degrade positive regulators of immunity. For example, the *P. syringae* T3SE HopBB1 promotes the degradation of the Arabidopsis transcription factor TCP14 [103]. TCP14 has been shown to contribute to ETI and also to be a repressor of JA-responsive genes [103,278]. HopBB1 mediates the physical association of TCP14 with the JA signaling repressor JAZ3, leading to the UPS-dependent degradation of both proteins, and subsequently the de-repression of JA responses [103]. The JA and SA pathways are mutually antagonistic, with SA limiting the growth of biotrophic pathogens and JA limiting the growth of necrotrophic pathogens. Therefore, the de-repression of JA responses benefits biotrophic pathogens, such as *P. syringae*, which are normally inhibited by SA [279]. The activity of HopBB1 is consistent with the activity of other *P. syringae* T3SEs, such as HopX1 and HopZ1a, which also activate the JA pathway to promote virulence (see Section 2 and Section 5). 

Similarly, the *P. syringae* T3SE HopM1 acts to promote UPS-dependent degradation of the Arabidopsis protein HOPM INTERACTOR 7 (AtMIN7) [108]. AtMIN7 is an ADP-ribosylation factor localized to the *trans*-Golgi network and early endosome which is required for both PTI and ETI [109]. Loss-of-function *atmin7* mutant plants are deficient in responses to the defense elicitors flg22 and benzothiadiazole as well as the T3SE AvrRpt2 [109]. As the N-terminus of HopM1 physically interacts with AtMIN7, and AtMIN7 is subsequently ubiquitinated and degraded, it is likely that HopM1 acts as an adaptor protein between AtMIN7 and components of the UPS [108]. Interestingly, there is also evidence that HopM1 acts as a proteasome inhibitor [157]. Transient expression of HopM1 in *N. benthamiana* resulted in decreased breakdown of the fluorogenic proteasomal activity reporter peptide Suc-LLVY-AMC, which suggests that HopM1 may serve dual roles in mediating host UPS activity [157]. Notably, this study identified several other *P. syringae* T3SEs as proteasome inhibitors, including HopAO1, HopA1, and HopG1. This suggests the existence of additional UPS-targeting T3SEs whose activities have yet to be characterized.

### 8.6. T3SEs That Indirectly Affect Host Protein Phosphorylation—AvrB, AvrRpm1, and AvrE-Type T3SEs

In the case of AvrB and AvrRpm1, pathogen virulence is influenced by indirectly facilitating the phosphorylation of host proteins. Bacterial delivery of either AvrB or AvrRpm1 results in the phosphorylation of RIN4, which is recognized by the NLR RPM1 to elicit ETI [25,90]. In terms of virulence function in the absence of RPM1, one regulatory mechanism demonstrated for AvrB involves direct interactions with SGT1 and RAR1 in a complex that also includes Hsp90 [91,280]. Within this complex, both AvrB and Hsp90 bind MAP KINASE 4 (MPK4) to facilitate its activation via phosphorylation [91]. There is currently no evidence for direct phosphorylation of MPK4 by AvrB, so this T3SE may enhance the activity of upstream kinases such as MKK1 or MKK2. Nonetheless, activated MPK4 interacts with and phosphorylates RIN4, followed by RIN4-mediated activation of the plasma membrane H+-ATPases AHA1 and/or AHA2, which stimulates the degradation of negative regulatory proteins from the JAZ family [91,281,282,283]. Ultimately, AvrB acts to upregulate JA signaling pathways, increase stomatal apertures, and suppress PTI in *rpm1* mutant plants [90,91,283]. In addition, a RIN4 immunoprecipitation experiment in AvrRpm1-expressing plants identified the RLCK RIPK as an additional RIN4-interacting kinase [90]. Five additional RLCKs were later identified that also interact with RIN4, AvrB, and each other [284]. All of these RLCKs catalyze RIN4 phosphorylation, and the addition of a phosphate group at threonine-166 appears to be the most relevant modification for both the virulence and avirulence functions of AvrB [90,284]. It is likely that AvrRpm1 functions in a similar manner to mediate RIN4 phosphorylation.

The AvrE family of T3SEs also indirectly modulates host protein phosphorylation. Members of this family are found in a variety of phytopathogenic bacteria, including *Pseudomonas* (AvrE1), *Ralstonia* (PopS), *Erwinia* (DspA/E), *Dickeya* (DspE), *Pantoea* (WtsE), and *Pectobacterium* (DspE) species [285]. While there is some variation among different host–pathogen combinations, AvrE-type T3SEs contribute significantly to the development of water-soaked lesions and tissue necrosis and may promote pathogen virulence through the suppression of immune responses such as callose deposition [286,287,288,289,290,291,292]. The amino acid sequences of AvrE family members frequently contain two conserved features: a WxxxE motif and a putative C-terminal ER membrane retention/retrieval signal [285]. Both of these regions are required for the full virulence activity of AvrE1 and WtsE [293]. Despite the putative ER retention signal, AvrE1 is primarily localized to the plasma membrane as well as plasma membrane-associated vesicle-like structures, and its localization is not affected by the deletion of either the WxxxE motif or the ER retention sequence [294]. The WxxxE motif is also found in T3SEs from animal pathogens, and these T3SEs are thought to mimic host guanine nucleotide exchange factors [295]. While a direct enzymatic activity remains to be elucidated for AvrE-type T3SEs, a yeast two-hybrid screen revealed that WtsE interacts with Protein Phosphatase 2A (PP2A) subunit proteins [93]. In planta experiments also demonstrated that AvrE1 interacts with multiple PP2A B’ subunits, and that inhibition of PP2A with the chemical cantharidin delays cell death elicited by either AvrE1 or WtsE [93]. This suggests that AvrE-type T3SEs act to enhance the phosphatase activity of PP2As. The potential functional implications of this enhancement were suggested by the results of synthetic genetic array screens in which WtsE or DspA/E was expressed in a library of yeast gene deletion mutants [93,94]. Both of these screens indicated that WtsE and DspA/E disrupt sphingolipid biosynthesis in yeast by depleting sphingolipid precursor molecules [93,94]. More specifically, DspA/E appears to activate the PP2A Cdc55 in yeast to catalyze the dephosphorylation of Orm proteins, which then negatively regulate one of the initial steps in the sphingolipid biosynthetic pathway [94]. An analogous regulatory mechanism has not yet been demonstrated in plant cells, but given that sphingolipid metabolism impacts vesicular trafficking in plants [296,297], this pathway could represent a T3SE target for modulating PTI. Furthermore, the inhibition of sphingolipid metabolism in plants ultimately causes cell death [298,299], which may underlie the necrotic symptoms elicited by AvrE-type T3SEs. Finally, yeast two-hybrid analyses have suggested that WtsE and DspA/E interact with LRR-RLK proteins [93,300], although the impact of this interaction on LRR-RLK kinase activity and in planta bacterial growth is currently unknown.

### 8.7. T3SEs That Alter The Subcellular Localization of Host Proteins—HopI1

The virulence function of the *P. syringae* T3SE HopI1 involves subcellular relocalization of host proteins. The amino acid sequence of HopI1 contains a C-terminal region that resembles a DnaJ domain, which typically mediates interactions with the chaperone Hsp70 to regulate protein homeostasis [301,302]. Generally, DnaJ proteins deliver unfolded substrates to Hsp70, stimulate Hsp70 ATPase activity, and stabilize interactions with the unfolded client protein as part of a co-chaperone complex [302]. In agreement with this behavior, HopI1 interacts with an Arabidopsis Hsp70 ortholog in a DnaJ domain-dependent manner and stimulates its ATPase activity [107]. Furthermore, HopI1 relocalizes Hsp70 from a largely cytosolic distribution to a significant association with chloroplasts. While the specific virulence function of HopI1 remains to be determined, HopI1 localization to chloroplasts results in modifications to the ultrastructure of thylakoid grana, reduced SA accumulation, and suppression of SA-responsive gene expression [303]. The relocalization of Hsp70 to chloroplasts may facilitate this suppression as well as compromise other aspects of host immunity through reductions in the amount of active Hsp70 in the cytosol.

### 8.8. T3SEs That Affect Auxin Signaling—AvrRpt2

Another indirect manipulation of phytohormone signaling appears to be mediated by AvrRpt2. Transgenic Arabidopsis plants with constitutive AvrRpt2 expression exhibit phenotypes associated with altered auxin physiology, including longer primary roots, more lateral roots, enhanced sensitivity to exogenous auxin, and higher levels of endogenous free indole acetic acid (IAA) [304]. In planta co-expression of AvrRpt2 and auxin/IAA transcriptional repressor (AXR) proteins results in AXR degradation, similar to the observed response to exogenous auxin [101]. Intriguingly, AXR degradation and AvrRpt2-mediated induction of auxin-responsive genes require an intact cysteine protease catalytic triad in AvrRpt2, but AXR degradation is reduced by treatment with the proteasome inhibitor MG132. This suggests that AvrRpt2 may stimulate auxin signaling by cleaving a yet-unidentified protein that in turn regulates the proteasomal degradation of AXR proteins. Bacterially delivered AvrRpt2 does enhance endogenous IAA accumulation, and exogenous auxin application increases the susceptibility of Arabidopsis to *P. syringae* infection [304], so AXR degradation represents an important virulence function for AvrRpt2.

### 8.9. T3SEs Impacting Reactive Oxygen Species Signaling—RipAK

Reactive oxygen species (ROS) also play an important role in immune signaling and may directly inhibit pathogen growth [305,306]. The T3SE RipAK from *R. solanacearum* is targeted to peroxisomes, and strains lacking this T3SE elicit a more rapid and stronger nonhost HR in tobacco, accompanied by greater ROS accumulation [115]. RipAK binds to and inhibits the activity of catalase enzymes that degrade hydrogen peroxide [115]. Transcriptional profiling of plants inoculated with *R. solanacearum* strains with or without RipAK indicates that this T3SE suppresses immunity during the early stages of infection, likely by interfering with ROS-mediated signaling [115].

### 8.10. Transcription Activator-Like Effectors (TALEs)

The host transcriptome represents an additional host–pathogen interface, and it can be manipulated by a class of T3SEs known as transcription activator-like effectors (TALEs) [116]. Although they are most well studied in *Xanthomonas* spp., TALE-like proteins have also been characterized in *R. solanacearum* and the bacterium *Mycetohabitans rhizoxinica*, which is an endosymbiont of the fungus *Rhizopus microspores* [307,308]. Typically, TALEs contain several conserved domains, including a T3SS translocation signal, a DNA-binding region, nuclear localization signals, and a transcriptional activation domain [309,310,311,312,313,314,315]. Binding to DNA is conferred by a series of tandem amino acid repeats each comprising 33 to 35 residues, within which the hypervariable residues at positions 12 and 13 (the repeat variable diresidues) facilitate binding to specific nucleotide base pairs in the promoters of target genes [316,317,318,319]. Transcriptional activation by TALEs involves interactions between the TALE activation domain and the gamma subunit of the plant transcription factor TFIIA [320,321]. Known TALE targets include genes that encode sugar transporters, abscisic acid biosynthetic enzymes, transcription factors, and sulfate transporters [322,323,324,325,326]. Given that the upregulation of these genes enhances susceptibility to infection, mutations within the effector binding site may eliminate TALE activity, although some effectors interact with conserved sequence motifs, such as TATA boxes, that cannot be altered without losing their native function in plants [327,328]. Alternatively, the effects of TALEs can be alleviated by mutations in the TFIIA gamma subunit that no longer bind the TALE activation domain, by “decoy” promoters that trap TALEs into activating cell death-promoting “executor” genes, or by NLR-mediated ETI [329,330,331,332]. On the other hand, truncated TALEs lacking the activation domain were recently found to interfere with NLR-mediated ETI, likely as dominant negative NLR interactors [333,334]. Overall, TALEs are important players in the evolutionary dynamics of several host–pathogen interactions.

## 9. T3SEs with Predicted Enzymatic Activities

Although the structural and sequence features of T3SE proteins can provide hints about their potential enzymatic functions, it may be difficult to obtain conclusive biochemical evidence of such activities. One example of this obstacle is HopAF1, which is a broadly distributed T3SE found in *Pseudomonas*, *Xanthomonas*, *Ralstonia*, and *Acidovorax* species [87]. Structural homology modeling identified a region of HopAF1 with similarity to deamidase enzymes. These enzymes replace amide side chain groups with a carboxylate group to irreversibly convert asparagine residues to aspartic acid and glutamine to glutamic acid [335]. The amino acid sequence of deamidase proteins includes conserved cysteine and histidine catalytic residues, which are also found in the deamidase-like region of HopAF1 [87]. Transgenic expression of HopAF1 in Arabidopsis enhanced the in planta growth of a *P. syringae* pv. *tomato* DC3000 T3SE polymutant (D28E) and ameliorated the flg22-induced inhibition of bacterial growth, suggesting that HopAF1 suppresses PTI [87]. Importantly, the putative cysteine/histidine catalytic residues were required for this suppression. Yeast two-hybrid screening, as well as subsequent co-immunoprecipitation and BiFC assays, indicated that HopAF1 interacts with a methylthioadenosine nucleosidase (MTN1) and its paralog MTN2 [87]. Methylthioadenosine nucleosidases participate in a methionine recycling pathway to maintain methionine as a feedstock for ethylene biosynthesis, which increases following PTI activation [336,337,338]. Inoculation of Arabidopsis leaves with *Pseudomonas fluorescens* elicits PTI and ethylene production, while ethylene is not elevated in a *mtn1mtn2* double mutant, nor when *P. fluorescens* expresses HopAF1 [87]. Mass spectrometry analyses did not detect any modified amino acids in MTN1 or MTN2 in the presence of HopAF1, but deamidation-mimicking substitutions in both MTN1 and MTN2 resulted in a loss of enzymatic function [87]. As such, it is likely that HopAF1 inhibits MTN1 and MTN2 to reduce ethylene biosynthesis and, in turn, suppress PTI, although the precise mechanism remains to be resolved. It is worth noting, however, that multiple pathogens of other eukaryotic hosts utilize T3SEs with deamidase activity to promote their virulence [335].

Manipulation of phytohormone signaling is also central to the virulence function of the chloroplast-localized *R. solanacearum* T3SE RipAL. Sequence analysis revealed that RipAL bears the hallmarks of a class III lipase enzyme, including a catalytic triad of serine/aspartic acid/histidine with the serine located within a conserved GXSXG motif [88]. Transient expression of RipAL in *N. benthamiana* inhibits flg22-triggered responses such as a ROS burst and PTI marker gene expression, and this inhibition is dependent on the putative catalytic residues of HopAF1 [88]. The transient expression of RipAL also stimulates increased JA and JA-Ile levels in leaves and induction of JA signaling marker genes, matched by reduced SA accumulation and lower expression of SA marker genes, again in a RipAL catalytic site-dependent manner [88]. Mechanistically, RipAL is similar to known lipases such as Arabidopsis DEFECTIVE IN ANTHER DEHISCENCE1 (DAD1), which act on cellular lipids to release linoleic acid as an important precursor for JA biosynthesis [339,340,341]. The primary barrier to validating RipAL lipase activity appears to be the inability to recover soluble purified protein for in vitro assays [88]. Nonetheless, the requirement for the putative lipase catalytic residues of RipAL for JA induction and pathogen virulence promotion strongly suggests a role for lipase activity. The strategy of suppressing SA signaling by upregulating JA signaling is also well established with the JA-mimicking virulence factor coronatine [342].

## 10. Context Matters: T3SE Activation in Eukaryotic Hosts

Upon delivery into host cells, several T3SE proteins are activated by interactions with endogenous enzymes or small molecules as a prerequisite for their contribution to pathogen virulence. The covalent attachment of fatty acids to specific residues within a protein (acylation) is the most common host-mediated modification of T3SE proteins [343]. Myristoylation involves the formation of an amide linkage between myristic acid (a 14-carbon saturated fatty acid) and the α-amino group of an N-terminal glycine residue. If the glycine is the second residue in a protein, it can be exposed by methionine aminopeptidase enzymes [344], while glycines at other locations can be exposed by proteolytic cleavage of N-terminal amino acids, as seen with AvrPphB [345]. Proteins can also be modified by S-palmitoylation through the attachment of palmitic acid (a 16-carbon saturated fatty acid) to the thiol side chain of a cysteine residue. While both of these hydrophobic modifications can anchor proteins in membranes, S-palmitoylation confers a stronger membrane association [344]. Much of the evidence for T3SE acylation is indirect, and myristoylation is presumed based on the loss of virulence or avirulence function in glycine-to-alanine substitution mutants of AvrPto, AvrRxo1, HopF2, HopO1-1, HopZ1a, HopZ4, and XopJ [32,46,193,346,347,348,349]. Additional substitutions at cysteine residues suggest that AvrRpm1, HopAF1, and HopZ1b are also S-palmitoylated near the N-terminus [87,350,351], and AvrPto is predicted to undergo palmitoylation as well [350]. Radiolabeled myristic acid was used to more directly detect in planta myristoylation of AvrB and AvrRpm1 [350]. A similar experiment using radiolabeled fatty acids showed that AvrPphB undergoes both myristoylation and S-palmitoylation in yeast [345]. These modifications significantly influence the subcellular localization of these T3SEs, and it is likely not coincidental that many of the targets of these effectors are also membrane-localized (Table 1). From an evolutionary perspective, myristoylation appears to be an exclusively eukaryotic modification [352], although prokaryotes do produce methionine aminotransferases [353]. The myristoylation consensus sequence contains an invariant glycine with some restrictions on the amino acid composition at adjacent positions, which can impact the efficacy of the methionine aminotransferase and/or acylation reactions [354]. This sequence may have been encoded within genes obtained through horizontal transfer or may have emerged through mutation and selection over time.

A second group of T3SEs is phosphorylated in host cells, although this modification has variable impacts on effector activity. The full virulence function of AvrPtoB requires in planta phosphorylation at serine-258, which is catalyzed by the Snf1-related kinase SnRK2.8 [355,356]. This can be counteracted, however, by the interaction of AvrPtoB with either Pto kinase or the lectin receptor-like kinase LecRK-IX.2, which phosphorylate AvrPtoB at threonine-450 or serine-335, respectively, to compromise the virulence-promoting E3 ubiquitin ligase activity of the T3SE [357,358]. For AvrPto, phosphorylation at serine-147 and serine-149 is required for its full virulence-promoting activity on susceptible tomato plants [359,360]. These modifications are also required for AvrPto avirulence in certain *Nicotiana* species but appear to be dispensable for Pto-mediated ETI in tomato. In contrast to AvrPtoB, AvrPto phosphorylation occurs independently of Pto kinase. Another phosphorylated T3SE is AvrB, shown to be a substrate of RIPK in vitro [90]. The specific phosphorylation site(s) and their functional implications have not been thoroughly characterized, although a non-phosphorylatable AvrB mutant cannot elicit RPM1-mediated ETI [361]. In a similar fashion, the phosphorylation of AvrBsT by PIK1 enhances the AvrBsT-elicited HR in *N. benthamiana* [92]. Finally, HopQ1, XopN, XopQ, and XopX rely on phosphorylation to varying degrees to facilitate interactions with their 14-3-3 protein targets [110,117,119,121]. Notably, HopQ1 can be phosphorylated by multiple calcium-dependent protein kinases and Snf1-related kinases in vitro [269]. Given that 14-3-3 proteins act as intracellular monitors of client protein phosphorylation status [265], these T3SEs may have evolved to use phosphorylation as a decoy to trap their protein targets.

In addition to phosphorylation, ubiquitination also regulates the activity of AvrPtoB. This T3SE appears to autoubiquitinate in vitro at multiple lysine residues in the C-terminal region [165,362]. Alleles of AvrPtoB with several lysine-to-arginine substitutions exhibit significantly less ubiquitin binding and are unable to suppress ETI. These mutants also lose E3 ubiquitin ligase activity, although the precise role of ubiquitin binding in this context remains to be elucidated.

Host-mediated proteolytic cleavage underlies the activation of AvrRps4 in planta. This processing does not occur in bacteria or yeast cells, indicating that a plant host protease is required [363]. Cleavage occurs between glycine-133 and glycine-134, releasing a chloroplast-localized N-terminal fragment that promotes pathogen virulence and a C-terminal fragment that is sufficient to elicit RRS1/RPS4-mediated ETI [363,364,365]. Interestingly, this cleavage is required for the virulence function of AvrRps4 but is not necessary for avirulence [363].

The activation of AvrRpt2 involves an association with a eukaryotic cyclophilin peptidyl-prolyl cis-trans isomerase [366]. In Arabidopsis, the cyclophilin ROC1 binds AvrRpt2 at four GPxL motifs and catalyzes the folding of this T3SE by prolyl isomerization [367]. Once folded, AvrRpt2 undergoes self-cleavage to release a 71 amino acid fragment from the N-terminus, and the activated T3SE can then proteolytically cleave host targets such as RIN4 [126,368]. The requirement for AvrRpt2 activation by a eukaryotic factor may have evolved to avoid the presence of potentially detrimental protease activity in the bacterial cytosol.

A similar strategy may underlie the eukaryotic host factor-dependent activation of other T3SEs. Binding of phytic acid (inositol hexakisphosphate) to PopP2 and HopZ1a induces conformational changes that allosterically enhance acetyl-CoA binding and thus increase the acetyltransferase activity of these T3SEs [369,370]. The activation of HopZ3 by phytic acid is implied by the loss of acetyltransferase activity in HopZ3 mutants bearing amino acid substitutions at putative phytic acid binding sites [198]. The calcium sensing protein calmodulin is unique to eukaryotes and is required as a co-factor for HopE1 [104]. This feature may provide an additional layer of spatiotemporal control by limiting HopE1 activity to cells where an immune response has been induced, which is associated with a major upregulation of Ca^2+^/calmodulin signaling [371]. The host cell environment is also sensed by RipAY, which preferentially binds to cytosolic *h*-type thioredoxins as a necessary precursor to RipAY activation [83,255]. Importantly, *h*-type thioredoxins are encoded by pathogen-inducible genes and mediate immune responses through nitric oxide signaling pathways [372,373]; therefore, they represent a logical stimulus for T3SEs with immunosuppressive functions.

## 11. Concluding Remarks

The T3SE repertoires of bacterial phytopathogens comprise a diverse toolkit for the manipulation of host immunity. A large number of T3SEs are dedicated to the suppression of PTI by targeting PAMP perception and downstream kinase signaling cascades, cytoskeleton-mediated transport of defense-related cargo, phytohormone biosynthesis, and signaling pathways, as well as host gene expression. Effector-triggered immunity is also targeted, and the evolutionary dynamics of plant–microbe interactions are aptly illustrated by the deployment of T3SEs that interfere with the recognition of other T3SEs. Overall, there is remarkable convergence onto common groups of host targets, both within and across pathogen genera. This functional focus highlights the fundamental regulatory components of host immunity and potentially shows the power of horizontal gene transfer in the evolution of virulence-promoting proteins. It is also evident that T3SEs can be multifunctional tools capable of manipulating multiple targets using different mechanisms, such as with HopQ1 or AvrBsT. As T3SEs evolve, those with low specificity and several targets may be more resilient to the mutation or loss of a single virulence target, although these T3SEs must bind their targets with sufficient affinity to modify their substrate and/or compete with endogenous host proteins at common binding sites. There is also a risk that a broadly acting T3SE could inadvertently inactivate a negative regulator of immunity or modify a target in a manner that is recognized by a NLR. These opposing selective forces likely have, and continue to, shape the biochemical properties of bacterial T3SEs. 

A number of T3SEs remain to be thoroughly characterized regarding their mechanism of action and contribution to virulence, which can present several challenges. Sequence analyses may not reveal any obvious homologies to known enzymes, especially if there is limited amino acid conservation outside of a small number of active site residues. Protein structures can be extremely informative, but protein purification and/or crystallization may not be straightforward for certain T3SEs. Purification of T3SE proteins may also be an obstacle for in vitro enzyme activity assays, which would be further complicated if T3SE activity requires host-mediated modifications or additional host proteins. Some T3SEs provide subtle contributions to pathogen growth in planta, making demonstrations of T3SE virulence function more difficult. Nonetheless, plants expressing individual T3SEs have been widely used in assays of PTI and ETI outputs to provide a starting point for defining the site of action for a particular T3SE. Interactome screens and large-scale T3SE–host protein interaction datasets [272,374] are useful resources for identifying putative T3SE targets. Multiple -omics approaches can be used to pinpoint the activity of a specific T3SE in planta at the transcriptional, proteomic, and metabolic levels. These can be combined with appropriate catalytic site mutants to provide stronger evidence for the enzymatic activity of a given T3SE.

Explorations of pathogen virulence mechanisms have also yielded valuable insights into the molecular systems used by host plants to recognize invading microbes and coordinate immune responses. Many NLRs monitor a decoy or guardee protein to detect T3SE-mediated manipulations. Under selection to evade recognition, it is likely that mutations causing a loss of T3SE enzymatic activity will also compromise virulence function, so the recognition of this activity by NLRs may provide more durable resistance compared to recognition via binding T3SE proteins directly. As such, genes encoding decoys or guardees are important considerations in breeding for disease resistance. Furthermore, engineered decoys/guardees can expand the range of pathogens that are recognized by a single NLR [375,376]. Host defenses may also be enhanced by loss-of-function mutations in T3SE targets that negatively regulate immunity or that are activated by T3SEs to promote pathogen growth. These proteins are referred to as “susceptibility targets” and are of considerable interest as potential mediators of durable pathogen resistance, barring any detrimental effects on plant growth arising from the functional loss of such targets [377,378,379,380,381]. Overall, continued investigations of T3SE functions will address the need to “know thy enemy” and will help drive efforts to breed for durable resistance in agricultural crops.

## Figures and Tables

**Table 1:** Summary of biochemical activities and (a)virulence functions of type III-secreted effector proteins from phytopathogenic bacteria.

Activity	Effector	Pathogen	*In planta* Activation	Host Target(s)/Substrate(s)	Consequences of Activity for Pathogen Virulence/Avirulence	Ref.
Acetyltransferase	AvrBsT	*Xanthomonas euvesicatoria*	Phosphorylation	ACIP1	Suppresses PTI and ETI; acetylated ACIP1 forms aggregates whose association with microtubules may be altered	[17]
				Unknown	Suppresses AvrBs1-mediated ETI in a SnRK1-dependent manner	[18]
	HopZ1a	*Pseudomonas syringae*	Myristoylation, phytate binding	Tubulin	Decreases microtubule networks, disrupts secretory pathways, and suppresses cell wall-based immunity	[19]
				JAZ proteins	Promotes degradation of JAZ proteins and activates JA signaling, acetylation not shown	[20]
				ZED1	Activates ZAR1-mediated ETI	[21]
	HopZ3	*Pseudomonas syringae*	Phytate binding	AvrB, AvrRpm1, RIN4, RIPK	Suppresses RPM1-mediated ETI	[22]
	PopP2	*Ralstonia solanacearum*	Phytate binding	WRKY proteins	Prevents WRKY proteins from binding DNA and activating immunity genes	[23,24]
				RRS1	Activates RRS1/RPS4-mediated ETI	[23,24]
ADP-ribosyltransferase	AvrRpm1	*Pseudomonas syringae*	Myristoylation/Palmitoylation	RIN4	Promotes RIN4 phosphorylation, which enhances associations between RIN4 and exocyst subunits and dampens flg22-induced callose deposition; activates RPM1-mediated ETI	[25,26,27]
	HopF2	*Pseudomonas syringae*	Myristoylation	RIN4	Inhibits proteolytic cleavage of RIN4 by AvrRpt2 to prevent AvrRpt2-induced ETI	[28]
				MKK4, MKK5	Disrupts MAP kinase cascade to block PTI	[29,30]
				BAK1	Inhibits multiple downstream signaling events, including the phosphorylation of BIK1 and MAPKs, to block PTI	[31]
	HopO1-1 ^1^	*Pseudomonas syringae*	Myristoylation	PDLP7	Destabilizes PDLP7 to increase plasmodesmata-mediated molecular flow and enhance bacterial spread within tissues	[32]
	(HopO1 allele)			Unknown	Activates ZAR1-mediated ETI	[33,34]
	HopU1	*Pseudomonas syringae*	---	GRP7	Interferes with GRP7 binding to PRR transcripts, thus reducing PTI	[35,36]
Cysteine protease	AvrPphB	*Pseudomonas syringae*	Myristoylation/Palmitoylation	BIK1 and other PBS1-like protein kinases	Suppresses PTI	[37]
				RIPK	Blocks the recognition of AvrB by RPM1	[38]
				PBS1	Activates RPS5-mediated ETI	[39,40]
	AvrRpt2	*Pseudomonas syringae*	Folding assisted by host cyclophilin ROC1	RIN4	Activates RPS2-mediated ETI, but suppresses PTI in plants lacking RPS2; blocks recognition of AvrRpm1 and AvrB by RPM1	[41,42,43]
	HopN1	*Pseudomonas syringae*	---	PsbQ	Reduces pathogen-induced ROS production and necrotic disease symptoms	[44]
	HopX1	*Pseudomonas syringae*	---	JAZ proteins	Enhances JA signaling, repressing SA signaling and promoting susceptibility to (hemi)biotrophic pathogens	[45]
	HopZ4	*Pseudomonas syringae*	Myristoylation	RPT6, proteasome	Inhibits proteasome activity	[46]
	RipE1	*Ralstonia solanacearum*	---	JAZ proteins	Enhances JA signaling, repressing SA signaling and promoting susceptibility to (hemi)biotrophic pathogens	[47]
	XopJ	*Xanthomonas euvesicatoria*	Myristoylation	RPT6, proteasome	Inhibits and degrades RPT6, suppresses cell wall-based immunity and SA signaling	[48,49]
						
SUMO protease	AvrXv4	*Xanthomonas euvesicatoria*	---	Unknown	Elicits a hypersensitive response in *N. benthamiana*	[50]
	XopD	*Xanthomonas euvesicatoria*	---	SlERF4	Destabilizes SlERF4 and suppresses ethylene-mediated defense responses	[51,52]
Threonine protease	HopB1	*Pseudomonas syringae*	---	BAK1	Suppresses PTI	[53]
Protein kinase	AvrRxo1	*Xanthomonas* spp. (and *Acidovorax, Burkholderia*)	Myristoylation	NAD	Production of 3′-NADP (uncharacterized mechanism of virulence promotion)	[54,55]
				SINAT4	Enhances degradation of RD21A which suppresses drought-induced immunity	[56]
	HopBF1	*Pseudomonas syringae*	---	Hsp90	Inactivates Hsp90 to destabilize NLRs and dampen ETI; may also promote cell death during the necrotrophic phase of *P. syringae* infection	[57]
	XopAU	*Xanthomonas euvesicatoria*	---	MKK2	Activates MKK2 to manipulate MAPK signaling and promote chlorosis	[58]
Phosphothreonine lyase	HopAI1	*Pseudomonas syringae*	---	MPK3, MPK6	Disrupts MAPK signaling cascade and suppresses PTI	[59]
				MPK4	Activates SUMM2-mediated ETI	[60]
Tyrosine phosphatase	HopAO1	*Pseudomonas syringae*	---	EFR, LORE, and possibly FLS2	Suppresses PTI	[61,62]
Ubiquitin–proteasome machinery	AvrPtoB	*Pseudomonas syringae*	Phosphorylation/Ubiquitination	EFR, FLS2, BAK1, CERK1	Degrades PRRs to suppress PTI	[63,64,65]
				NPR1	Degrades NPR1 to disrupt SA-mediated signaling and SAR	[66]
				Fen	Degrades Fen to block AvrPtoB-mediated ETI	[67]
				Pto	Activates Pto/Prf-mediated ETI (Pto is neither ubiquitinated nor degraded)	[68,69]
	GALA proteins	*Ralstonia solanacearum*	---	SKP1-like proteins	Promotes virulence via an unknown mechanism	[70,71]
	RipAR	*Ralstonia solanacearum*	---	Unknown	Suppresses PTI	[72]
	RipAW	*Ralstonia solanacearum*	---	Unknown	Suppresses PTI	[72]
	RipV2	*Ralstonia solanacearum*	---	Unknown	Suppresses PTI	[73]
	XopAE	*Xanthomonas euvesicatoria*	---	Unknown	Suppresses PTI	[74]
	XopI	*Xanthomonas oryzae* pv. *oryzae*	---	OsTrxh2	Degradation of OsTrxh2 represses OsNPR-mediated signaling and SAR	[75]
	XopK	*Xanthomonas oryzae* pv. *oryzae*	---	OsSERK2	Virulence promotion	[76]
	XopL	*Xanthomonas euvesicatoria*	---	SH3P2	Suppresses PTI and promotes virulence by inhibiting autophagy	[77,78]
Uridylyltransferase	AvrAC	*Xanthomonas campestris* pv. *campestris*	---	BIK1, RIPK	Suppresses PTI and RPM1-mediated ETI	[79]
				PBL2	Activates ZAR1-mediated ETI	[80]
Phytase/Phosphatase	XopH	*Xanthomonas euvesicatoria*	---	Phytate	Suppresses PTI, activates Bs7-mediated ETI	[81]
Trehalose phosphate synthase	RipTps	*Ralstonia solanacearum*	---	Glucose-6-phosphate	Promotes trehalose synthesis (uncharacterized impact on virulence)	[82]
Gamma-glutamyl cyclo-transferase	RipAY	*Ralstonia solanacearum*	Thioredoxin binding	Glutathione	Suppresses PTI	[83]
Nudix hydrolase	RipN	*Ralstonia solanacearum*	---	ADP-ribose, NADH	Suppresses PTI	[84]
Nucleoside hydrolase	HopQ1	*Pseudomonas syringae*	Phosphorylation	Cytokinin precursors	Increases cytokinin accumulation to suppress PTI	[85]
	XopQ	*Xanthomonas* spp.	Phosphorylation	Natural substrate unknown	Contributes to pathogen virulence, although the role of nucleoside hydrolase activity is unknown	[86]
Deamidase (predicted)	HopAF1	*Pseudomonas syringae*	Myristoylation/Palmitoylation	MTN1, MTN2	Inhibits MTN activity to reduce ethylene biosynthesis and suppress PTI	[87]
Lipase (predicted)	RipAL	*Ralstonia solanacearum*	---	Chloroplast lipids (predicted)	Stimulates JA biosynthesis, repressing SA signaling and promoting susceptibility to (hemi)biotrophic pathogens	[88]
Indirect/Non-enzymatic mechanisms	AvrB	*Pseudomonas syringae*	Phosphorylation/Myristoylation	RIPK and other RLCKs	Enhances kinase activity to phosphorylate RIN4 and suppress PTI; can trigger ETI through RPM1	[89,90]
				MPK4	Upregulates jasmonate signaling to enhance pathogen susceptibility	[91]
	AvrBsT	*Xanthomonas euvesicatoria*	Phosphorylation	SGT1 homologs	Activates AvrBsT-mediated ETI	[92]
	AvrE-type T3SEs	*Pseudomonas syringae* (AvrE), *Pantoea stewartii* subsp. *stewartii* (WtsE), *Erwinia amylovora* (DspA/E)	---	PP2A B’ subunits	Enhances phosphatase activity, which may disrupt sphingolipid biosynthesis	[93,94]
	AvrPto	*Pseudomonas syringae*	Phosphorylation/Myristoylation/Palmitoylation	FLS2, EFR, CERK1, BAK1/SERK3	Suppresses PTI	[95,96]
				Pto	Activates Pto/Prf-mediated ETI	[97]
				SOBIR1	Suppresses Cf-4-mediated ETI	[98]
	AvrRpm1	*Pseudomonas syringae*	Myristoylation/Palmitoylation	RIPK and other RLCKs	Enhances kinase acitivity to phosphorylate RIN4 and suppress PTI; activates RPM1-mediated ETI	[89,90]
	AvrRps4	*Pseudomonas syringae*	Host-mediated proteolytic cleavage	BTS	Enhances apoplastic iron accumulation to promote pathogen proliferation	[99]
				RRS1	Activates RRS1/RPS4-mediated ETI	[24,100]
	AvrRpt2	*Pseudomonas syringae*	Folding assisted by host cyclophilin ROC1	Aux/IAA proteins, e.g., AXR2, AXR3	Enhances auxin signaling to promote susceptibility to infection	[101]
	AvrRxv	*Xanthomonas euvesicatoria*	---	ARI1 (14-3-3 protein)	Activates AvrRxv-mediated ETI	[102]
	HopBB1	*Pseudomonas syringae*	---	TCP14, JAZ3	Colocalizes TCP14 and JAZ3 for SCFCOI1-dependent degradation, de-repressing JA signaling and suppressing SA signaling	[103]
	HopE1	*Pseudomonas syringae*	Calmodulin binding	MAP65	Sequesters MAP65 away from the microtubule network, inhibits extracellular secretion of PR-1	[104]
	HopG1	*Pseudomonas syringae*	---	Kinesin	Promotes actin filament bundling and enhances symptom development; suppression of PTI	[105,106]
	HopI1	*Pseudomonas syringae*	---	Hsp70	Hsp70 is recruited to chloroplasts and forms a complex that suppresses defense responses	[107]
	HopM1	*Pseudomonas syringae*	---	AtMIN7	Facilitates AtMIN7 degradation to suppress PTI and ETI	[108,109]
	HopQ1	*Pseudomonas syringae*	Phosphorylation	14-3-3 proteins	Suppresses PTI	[110]
	HopW1	*Pseudomonas syringae*	---	Actin	Promotes virulence by disrupting actin filaments	[111]
	RipAC	*Ralstonia solanacearum*	---	SGT1 homologs	Disrupts SGT1–RAR1 interactions and blocks SGT1 phosphorylation to suppress ETI	[112,113]
				PUB4	Blocks PUB4 E3 ubiquitin ligase activity to suppress PTI	[114]
	RipAK	*Ralstonia solanacearum*	---	Catalases	Interacts with and inhibits catalases to interfere with reactive oxygen species-mediated signaling	[115]
	TAL effectors	*Xanthomonas* spp., *Ralstonia solanacearum*	---	DNA, often at gene promoters	Manipulate host gene expression to promote pathogen growth; may elicit ETI in specific host genetic backgrounds	Reviewed in [116]
	XopN	*Xanthomonas euvesicatoria*	Phosphorylation	TARK1, 14-3-3 proteins (e.g., TFT1)	Acts as a scaffold for TFT1–TARK1 interactions to suppress PTI	[117]
	XopP	*Xanthomonas oryzae pv. oryzae*	---	OsPUB44	Blocks OsPUB44 E3 ubiquitin ligase activity to suppress PTI	[118]
	XopQ	*Xanthomonas* spp.	Phosphorylation	14-3-3 proteins	Suppresses ETI (tomato) and PTI (rice)	[119]
				ROQ1	Activates ROQ1-mediated ETI	[120]
	XopX	*Xanthomonas oryzae pv. oryzae*	Phosphorylation	14-3-3 proteins	Suppresses PTI	[121]

^1^ ADP-ribosylation activity of HopO1-1 is inferred but not directly demonstrated.
